# Accelerated old basal ice loss in Tibetan glaciers after the 1990s

**DOI:** 10.1093/nsr/nwag558

**Published:** 2026-09-04

**Authors:** Baiqing Xu, Xiaolong Zhang, Tandong Yao, Wei Yang, Xiangke Xu

**Affiliations:** State Key Laboratory of Tibetan Plateau Earth System, Environment and Resources (TPESER), Institute of Tibetan Plateau Research, Chinese Academy of Sciences, Beijing 100101, China; State Key Laboratory of Tibetan Plateau Earth System, Environment and Resources (TPESER), Institute of Tibetan Plateau Research, Chinese Academy of Sciences, Beijing 100101, China; State Key Laboratory of Tibetan Plateau Earth System, Environment and Resources (TPESER), Institute of Tibetan Plateau Research, Chinese Academy of Sciences, Beijing 100101, China; State Key Laboratory of Tibetan Plateau Earth System, Environment and Resources (TPESER), Institute of Tibetan Plateau Research, Chinese Academy of Sciences, Beijing 100101, China; State Key Laboratory of Tibetan Plateau Earth System, Environment and Resources (TPESER), Institute of Tibetan Plateau Research, Chinese Academy of Sciences, Beijing 100101, China

**Keywords:** basal melt, glacier, Tibetan Plateau, proglacial lake sediment, radiocarbon

## Abstract

Surface meltwater that penetrates glacier interiors to the beds can warm the ice and enhance basal mass loss, exerting critical control over glacier dynamics. However, these processes remain poorly constrained in Tibetan Plateau glaciers, particularly those in the western sector, which have long been regarded as cold-based. Here, we used pollen radiocarbon ages from sediments in seven proglacial lakes, spanning catchments with 0.5%–55.0% glacier cover, to distinguish old terminal and/or basal ice melt from young surface snow/ice melt of upstream glaciers previously assumed to be cold-based (four lakes) and polythermal (three lakes) over the past 150 years. We show that after the 1990s, the melting of Tibetan glaciers shifted into a new regime, marked by accelerated mass loss of old basal ice under intensified surface melt conditions. These findings underscore the urgent need for integrated observations of both supra- and subglacial processes to resolve the evolving hydrothermal regimes in Tibetan glaciers under ongoing climate warming.

## INTRODUCTION

With climate warming, more surface meltwater can penetrate cold ice to the glacier bed via crevasses, moulins and/or hydrofractures under intensified melt conditions [1–3]. Even on unfractured surfaces, englacial conduits can form and reach the glacier bed through the ‘cut and closure’ mechanism, linking supra–subglacial drainage systems [4]. Surface meltwater delivered to the glacier bed can warm ice via cryohydrologic warming [5] and melt basal ice through viscous heat dissipation [6], thereby facilitating the evolution of subglacial hydrologic systems [1–3]. This process can drive a non-negligible mass loss in the basal ice. For instance, the average basal melt (5.2 ± 1.6 Gt per year from 1990 to 2010) [7] due to surface meltwater injection comprises ~8% of the surface mass loss (65 ± 18 Gt per year from 1992 to 2012) [8] for the Greenland Ice Sheet. Additionally, surface meltwater reaching the glacier base can increase subglacial water pressure, playing a crucial role in mountain glacier detachment [9]. These meltwater paths and their roles in glacier mass loss and dynamics have been extensively explored through observations and models in alpine temperate and high-latitude polythermal glaciers, as well as in the Greenland Ice Sheet [1–3,10,11].

Tibetan glaciers harbour the most extensive ice mass beyond the polar regions and are vital for sustaining water resources across Asia [12]. However, the spatio-temporal evolution of subglacial hydrologic systems remains poorly understood owing to limited field observations [13]. Glaciers in the cold, arid western Tibetan Plateau—encompassing the western Kunlun, Qiangtang, eastern Pamir, and western sectors of the Tanggula, Gangdis and Qilian ranges—have traditionally been considered predominantly cold-based [14], a thermal regime that is theoretically incompatible with subglacial drainage. However, evidence from the Aru glacier collapse in the inner Qiangtang Plateau [15], and surge-type glaciers in the western Kunlun [16] indicates polythermal basal conditions and deep penetration of surface meltwater into glacier beds. Complementary analyses of seasonal glacier dynamics over the past decade have revealed that evolving basal hydrology across extensive Tibetan glaciers drives pronounced autumnal velocity peaks [17]. Himalayan glacier modelling further suggests that under atmospheric warming, the transition from cold to temperate basal ice, estimated to occur over 100–150 years under heat advection–diffusion processes alone, can be accelerated to 50–60 years through crevasse-mediated meltwater infiltration [18]. Collectively, these findings demonstrate that supraglacial processes are already influencing basal melting, highlighting the urgent need for systematic investigations into the evolution of Tibetan glacier hydrological regimes.

Here, we explored the evolution of melting systems (surface versus basal melt) of Tibetan glaciers previously assumed to be cold-based and polythermal using proglacial lake sediments spanning the past 150 years. Since glacier melt-released materials ultimately deposit on the bottom of proglacial lakes, their sediments serve as excellent archives for studying the long-term continuous glacier melting status. The chosen temporal window is ideal for our enquiry: Following several centuries of climatic cooling during the Little Ice Age (LIA, 13th–19th century) [19,20], the Tibetan glaciers studied are expected to have retained substantial cold content [2,5,18,21]. At the onset of our study period, this legacy of cooling would have strongly limited the penetration of surface meltwater into the glacier interiors.

## RESULTS

We analysed sediments from seven proglacial lakes across contrasting climatic regimes on the Tibetan Plateau. These sites extend from the central–eastern Himalayas (Everest Lake and Qiangyong Co) and central Tanggula Mountain (Tanggula Lake) within the Indian monsoon domain, to the south-western Qiangtang Plateau (Cangmu Co and Dong Co) and western Kunlun (Aksai Chin and Guozha Co) within the westerly domain [22] (Fig. 1). Despite broadly uniform bedrock lithologies, the catchments occupy steep gradients in precipitation, cryospheric state and ecological conditions (Fig. 1 and Table S1 and Text S1).

**Figure 1.**
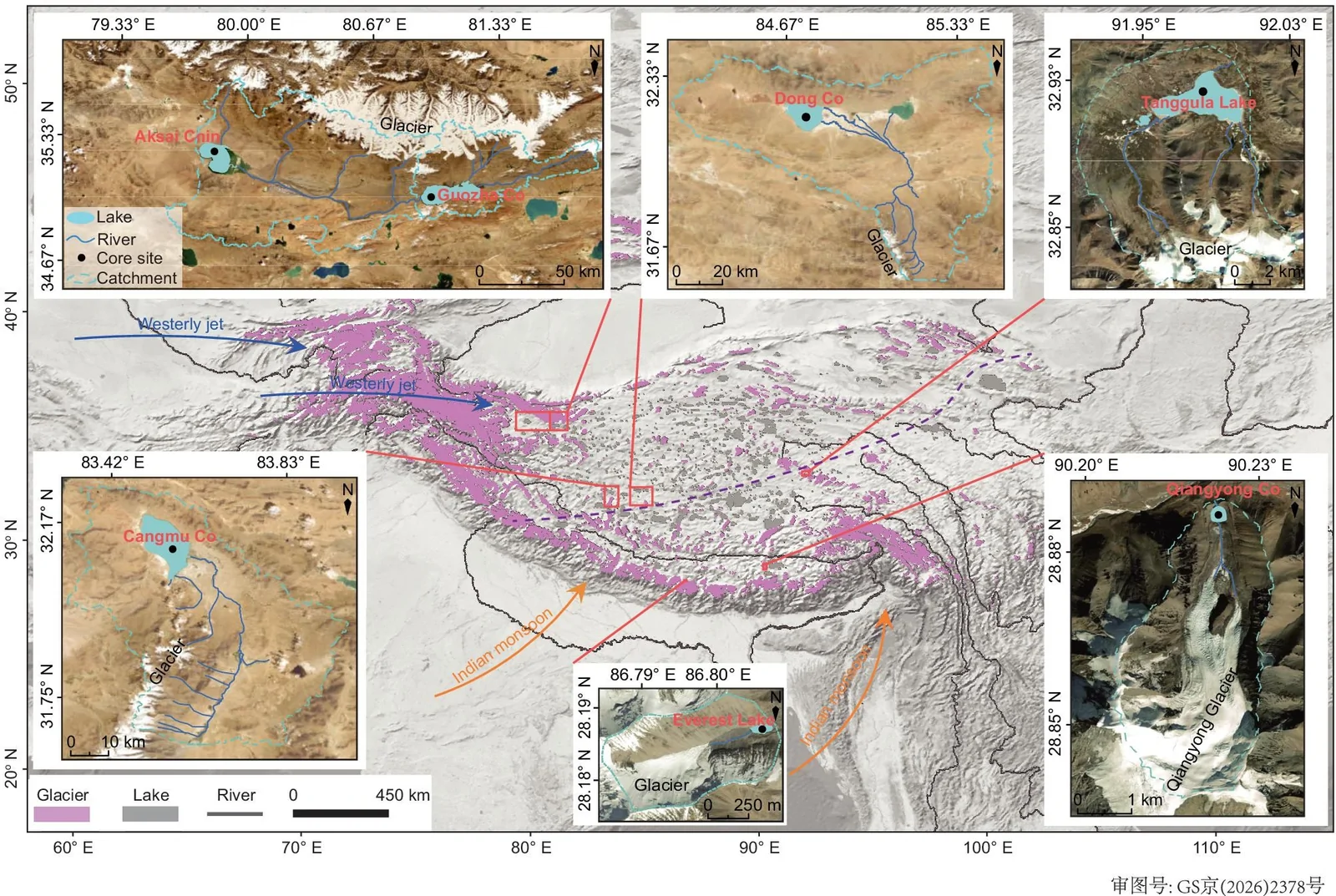
Locations of seven proglacial lake catchments on the Tibetan Plateau and their lake sediment core sites. The seven lakes are Aksai Chin, Guozha Co, Dong Co, Tanggula Lake, Cangmu Co, Everest Lake and Qiangyong Co. The locations are listed in Table S1. The figure also shows the distributions of glaciers, lakes and major Asian rivers. The dashed purple line represents the current boundary between the Indian monsoon and the westerly climate domains [22].

The mean annual precipitation (1979–2018) declines systematically from east to west, with regional averages of 440 mm in the monsoon domain, 250 mm in the south-western Qiangtang, and 170 mm in the western Kunlun. Upstream glaciers feeding the four lakes in the westerly domain are predominantly cold-based, whereas those sustaining the three monsoon-domain lakes are polythermal [14]. The catchment and lake areas spanned 1–5999 and 0.002–265 km^2^, respectively, with glacier coverage ranging from 0.5% to 55.0% (0.1–689 km^2^) and permafrost extent from 34.3% to 88.1% (1–6950 km^2^). Because runoff is generated at glacier termini and routed through extensive downstream permafrost zones before entering the lakes, we extended the conventional lake supply coefficient (catchment-to-lake area ratio) to derive two process-specific metrics: the glacier and permafrost meltwater supply coefficients, defined as the ratios of upstream glacier and permafrost areas to lake area. These metrics provide a physically grounded framework for evaluating the relative contributions of glacier- and permafrost-driven sediment delivery. By definition, the meltwater supply coefficients incorporate the influence of precipitation-driven surface erosion, and we further accounted for vegetation effects by calculating the catchment-averaged median annual maximum normalized difference vegetation index (NDVI_max_). Overall, across the seven lakes, the permafrost meltwater supply coefficients showed no systematic variations across the climate domains, whereas the glacier meltwater supply coefficients declined sharply in the monsoon domain (63.0 to 2.0) and more gradually (2.6 to 0.3) in the westerly domain. In parallel, NDVI_max_ generally increased with decreasing glacier meltwater supply coefficients in both domains (Fig. 2a and Table S1).

**Figure 2.**
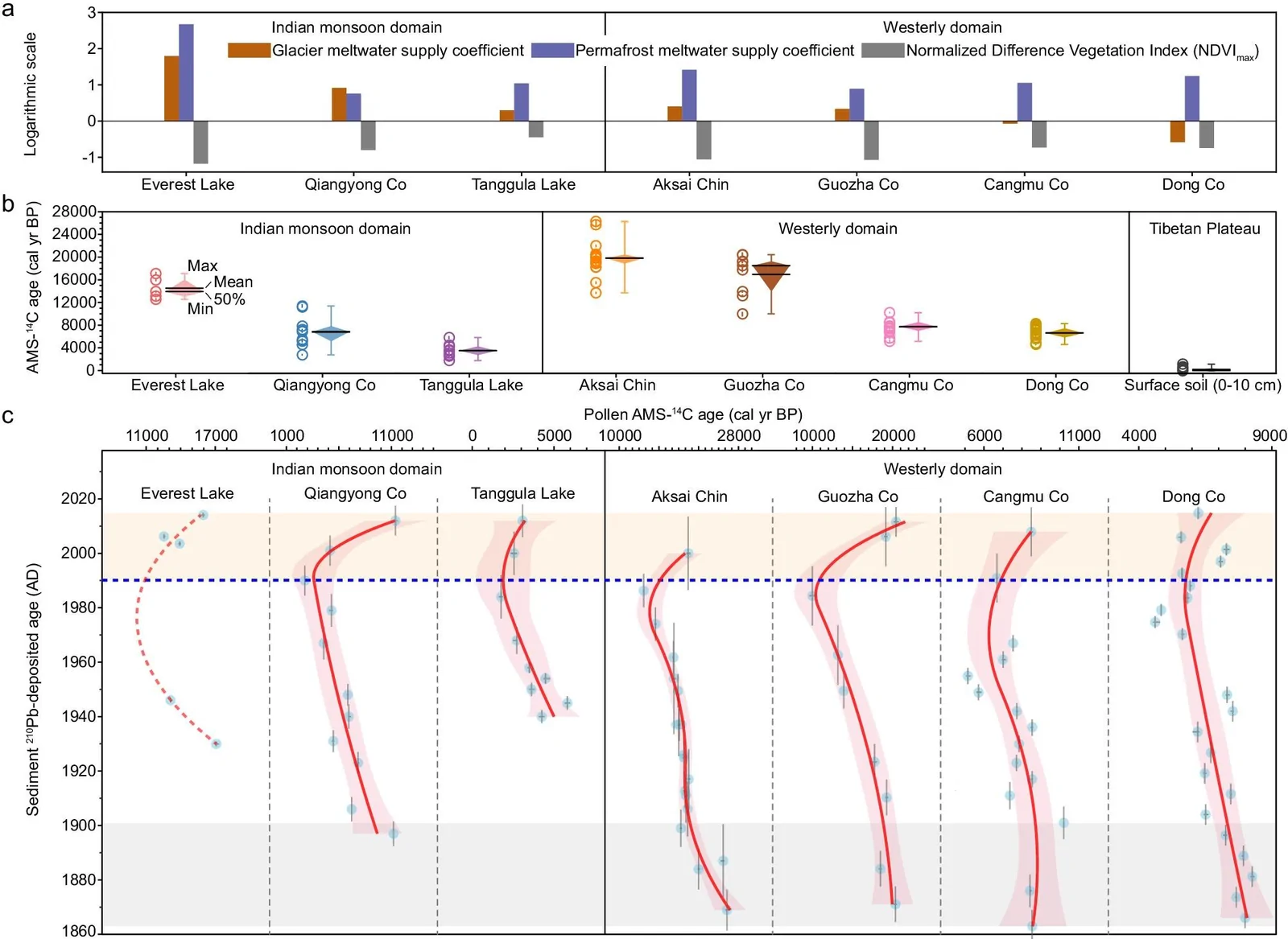
The glacier melting history over the past 150 years was reconstructed using pollen radiocarbon (AMS-^14^C) ages in the sediments of seven proglacial lakes. (a) NDVI_max_, together with glacier and permafrost meltwater supply coefficients, defined as the area ratios of glacier and permafrost to lake, respectively, for Everest Lake, Qiangyong Co and Tanggula Lake in the Indian monsoon climate domain, and for Aksai Chin, Guozha Co, Cangmu Co and Dong Co in the westerly climate domain. The *y* axis is presented on a logarithmic scale. (b) Box plots compare the pollen radiocarbon ages for each lake within their respective climate domains to the radiocarbon ages of soil organic matter from the 0–10 cm surface layers across the Tibetan Plateau [29]. (c) Temporal variations in pollen radiocarbon ages for each lake within their respective climate domains. Horizontal error bars, depicted as light grey lines, indicate two standard errors of pollen radiocarbon ages, with their size smaller than the symbols due to large age scales. Vertical error bars (light grey lines) denote the depths and age ranges of the lake sediments. The red curves represent quadratic B-spline fittings with 95% confidence bands (light rose-red shading). A detailed explanation of the spline-fitting methodology is provided in the Supplementary data. Three distinct glacier melt stages, highlighted by light grey, white and light orange shading bars, correspond to the specific periods depicted in Fig. 3. The dotted blue line indicates an abrupt shift in the melt paradigm of Tibetan glaciers during the 1990s, transitioning from intensified young surface ice melt to enhanced old basal ice melt.

In each lake, we retrieved one sediment core (except for two cores in Aksai Chin) and measured the lead (^210^Pb) and caesium (^137^Cs) isotopes in the sediments to determine their depositional ages. We ultimately chose the piecewise constant flux constant sedimentation rate (CFCS) model of ^210^Pb, establishing sediment depositional ages within the range of 1863–2017 for the seven lakes (Fig. S1 and Text S3). The sediment ^210^Pb-deposited ages were further verified by the ^137^Cs date markers of 1955, 1963 and 1986 (if present) for each lake (Fig. S1a–g). We extracted pollen grains from the sediments of each lake at intervals of 1–22 years and conducted microscopic analyses of selected pollen samples from five lakes to verify the overwhelmingly terrestrial vegetation origin of the extracted pollen grains (Figs S3 and S4 and Text S2). We ultimately analysed and used the accelerator mass spectrometry radiocarbon (AMS-^14^C) ages of all pollen samples for each lake as an efficient proxy to distinguish old terminal and/or basal ice melt from young surface snow/ice melt of upstream glaciers in the catchment. The theoretical basis is referred to in our previous work [23] and further improved as follows, as conceptually described in Fig. 3 and Fig. S5.

**Figure 3.**
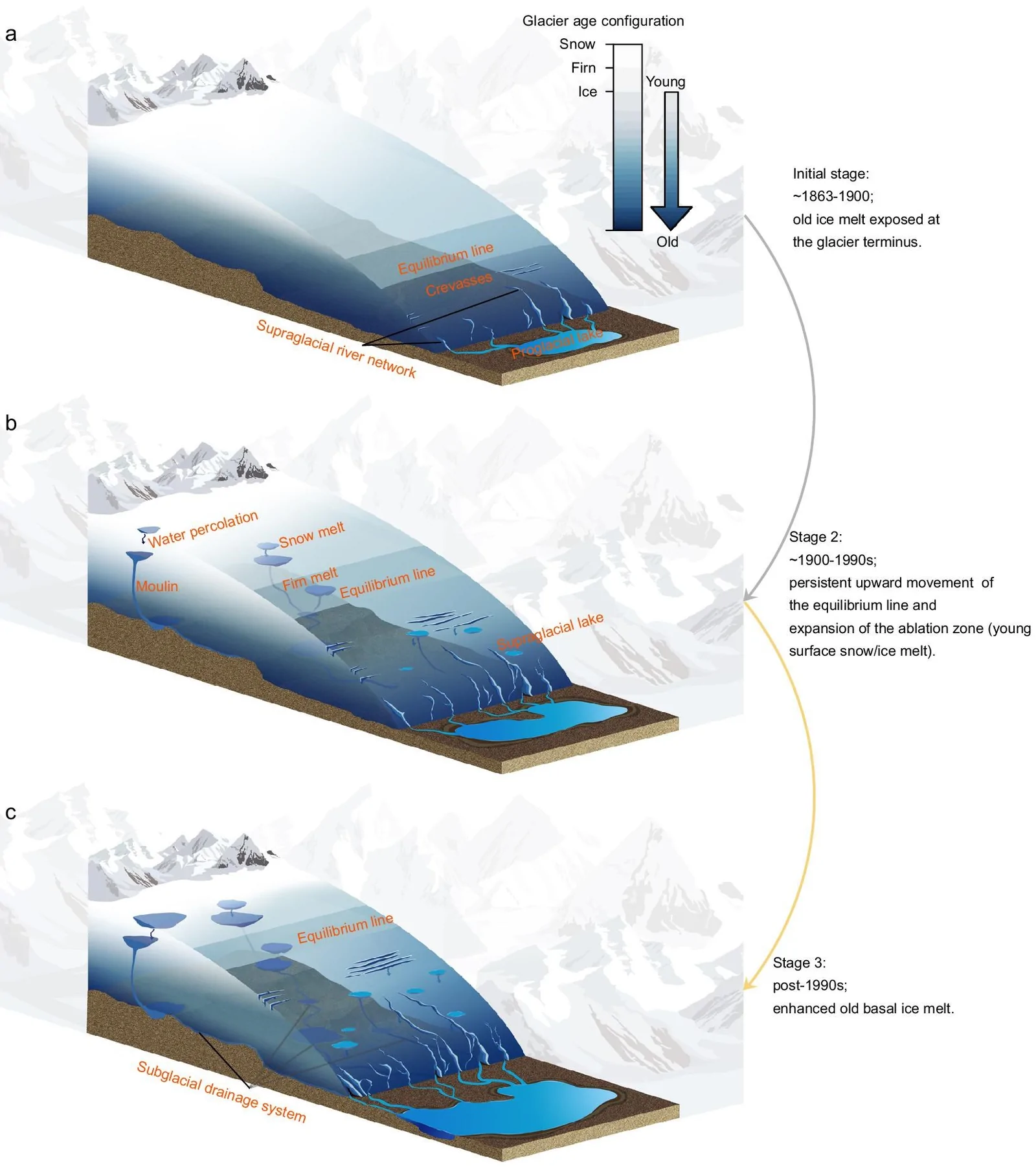
Schematic of glacier melting processes and age distribution of ice layers in a glacier. Three distinct melting stages [(a) ~1863–1900, (b) 1900–1990s and (c) post-1990s] were defined based on the data in Fig. 2c. Over time, as the equilibrium line and ablation zone have persistently shifted upward, relatively young surface meltwater has gradually been routed to the glacier bed (a, b), ultimately leading to the melting of old basal ice (c). This process resulted in an abrupt shift from decreasing to increasing pollen radiocarbon ages around the 1990s in sediments from the seven proglacial lakes (Fig. 2c).

The bedrock underneath the upstream glaciers in the seven catchments was uniformly composed of granite and metamorphic rocks (Table S1), and consequently, the materials produced by glacier-bed erosion contained no pollen. Therefore, pollen grains deposited in the seven lakes primarily originated from three pathways: (i) release of pollen entrained in glacier ice and snow during melt (glacier source), (ii) mobilization of pollen stored in downstream permafrost terrain through surface erosion and thaw-related runoff (permafrost source) and (iii) production and export of pollen from contemporary vegetation within each catchment (vegetation source).

Spatially, the radiocarbon ages of pollen grains in lakes within the Indian monsoon and westerly climate domains decreased markedly with decreasing glacier meltwater supply coefficients and increasing NDVI_max_, with average values ranging from ~3500 to 14 500 years and 6600 to 19 800 years, respectively (Fig. 2a and b and Table S2). The opposing correlations indicate that glacier- and vegetation-derived pollen exert contrasting roles in radiocarbon variations. To further quantify the relative source contributions, we applied multiple linear and ridge regression analyses using glacier and permafrost meltwater supply coefficients, and NDVI_max_ as predictors (Text S4). Analyses focused on post-2000-year observations to minimize temporal confounding and characterize present-day environmental conditions, thereby allowing spatial gradients to serve as analogues for temporal change (i.e. a space-for-time substitution [24]). The results derived from the full dataset are reported for comparison.

Regression analyses of post‑2000 observations reveal clear and opposing glacier vegetation controls across both climate domains (Fig. S6 and Table S3). In the westerly domain, ridge-stabilized standardized coefficients showed a pronounced positive glacier influence (+0.4908), countered by an almost equally strong vegetation-driven younging effect (−0.4614), while permafrost contributions remained weak and negative (−0.0404). In the Indian monsoon domain, glacier inputs continued to dominate (+0.2395), vegetation emerged under ridge penalization as a stable negative driver (−0.3889) and permafrost influence was minor and context-dependent. Together, these patterns demonstrate that glacier processes and vegetation production constitute the two principal—and opposing—controls on pollen radiocarbon age variations, whereas permafrost-derived inputs play a secondary role.

Nevertheless, we argue that the limited permafrost influence is largely embedded within vegetation-related surface erosion processes for the following three reasons: First, although thaw slumps or permafrost collapse can release deeply buried ancient pollen, their spatial footprints are small [25], and freeze–thaw erosion predominantly affects the shallow active layer (0–50 cm) [26,27]. Second, Δ^14^C measurements show that plant-derived carbon overwhelmingly dominates Tibetan Plateau topsoils [28], yielding young organic matter ages (average of ~200 years at 0–10 cm [29] and ~2570 years at 30–50 cm [28]), far younger than lake-derived pollen except at Tanggula (Fig. 2b and Table S2). Third, based on the highest sedimentation rates observed across the seven lakes (Fig. S1) and assuming spatially uniform deposition, we estimated basin-scale (excluding glacier and lake areas) erosion depths over the past 150 years to be <10 cm (0.1–9.3 cm). These constraints, combined with the regression results, indicate that variations in pollen radiocarbon ages are governed primarily by the age structure of pollen released from melting snow and ice in upstream glaciers, with permafrost processes acting mainly as a diluting influence. Similar glacier-dominated patterns have been documented for riverine organic matter in Gulf of Alaska watersheds with 0%–64% glacier coverage [30], providing strong external support for this interpretation.

Temporally, pollen radiocarbon ages across the seven lakes showed largely consistent variations (Fig. 2c and Text S5) despite their contrasting climate regimes, reinforcing the dominant age control of glacier melt-released pollen. Two interdependent lines of evidence explain this coherence. First, the Beijing Climate Center Earth System Model version 1 (BCC-ESM1) simulations indicated sustained increases in net primary productivity (NPP) across most lake catchments since 1850 (six lakes; Fig. S7), reflecting the long-term intensification of young pollen production. Second, the Tibetan Plateau has experienced a persistent bipolar moisture pattern since the mid-19th century, with progressively drier conditions in the Indian monsoon domain and wetter conditions in the westerly domain [31,32]. These opposing hydroclimatic trends should produce divergent surface erosion intensities across permafrost landscapes. If vegetation and permafrost processes were the primary drivers of pollen age, these contrasting erosion regimes would generate inconsistent temporal patterns among lakes. However, no such divergence was observed in this study.

Together, the spatial and temporal evidence indicates that the age structure of pollen entrained in glacier ice and snow is the principal determinant of lake-deposited pollen radiocarbon ages, whereas vegetation and permafrost processes contribute secondarily by diluting the signal with younger material without altering the dominant age structure.

For a given pollen grain released from glacier ice melt and ultimately deposited on the proglacial lake bottom, its radiocarbon age comprises two components: the time it resided in the glacier ice and the time it took for transportation to and residence in the lake sediments. Given that any melted ice layer and its released pollen grains have the same age, and because the studied lake sediments cover only 150 years of depositional history—within the error ranges of radiocarbon dating (Dataset S1)—the radiocarbon ages of the pollen in the seven lakes can approximately represent the ages of melted ice from the upstream glaciers at the time of sediment deposition, without needing to subtract the sediments’ ^210^Pb dates. In theory, the age distribution of ice layers in any glacier follows the general law of ice flow: Ice layers become older from the glacier surface to the base with depth and from the equilibrium line to the terminus along the surface [33] (Fig. 3). Changes in the pollen radiocarbon ages depend on the volume-mixed ratio of melted ice with different ages in upstream glaciers. Given that upstream surface and terminal and basal meltwater, respectively, bring younger and older pollen to the proglacial lake and supraglacial melt is always present, the pollen radiocarbon ages can be used to distinguish old terminal and/or basal ice melt from young surface snow/ice melt, as their relative contributions to glacier meltwater would lead to rejuvenation or aging.

## DISCUSSION

The pollen radiocarbon ages across the seven lakes showed broadly consistent variations over the past 150 years, shifting from a descending to an increasing tendency, with the latest turning point emerging in the early 1990s (Fig. 2c and Table S4). The timing of this shift varied spatially among the lakes, with most transitions occurring between the 1970s and 1990s. These differences largely reflect the heterogeneous temporal resolutions of the pollen samples (1–22 years; Dataset S1), which can blur or displace the apparent onset of the trend reversal. Alternatively, the offsets may indicate spatially distinct glacier responses to climate forcing; however, this possibility cannot be robustly evaluated within the constraints of the current sampling resolutions.

We divided the evolution of pollen radiocarbon ages into three stages. In the initial stage, from ~1863 to 1900, the pollen radiocarbon ages were generally at their maximum, with stable or decreasing trends (Fig. 2c). We attribute this pattern to the dominant contribution of surface old ice melt exposed at the glacier terminus to glacial meltwater, as the equilibrium line at the end of the LIA was at its lowest (Fig. 3a).

During the second stage, from ~1900 to the 1990s, the pollen radiocarbon ages persistently decreased from maximum to almost minimum values in the seven lakes (Fig. 2c). These declining trends are best explained by the upward expansion of the ablation zone and the associated increase in meltwater production from upstream young surface snow/ice (Fig. 3b and Fig. S5), which was largely in line with climate warming since the late 19th century [34]. This gradually reduces the relative proportion of meltwater contribution from the old ice exposed at the retreating glacier terminus [19,20]. As shown in Fig. S5a, the surface mass balance of mountain glaciers typically increases linearly with altitude [33]. The altitudinal mass balance gradients in the ablation area (ablation gradients) can potentially remain unchanged [35], increase [36,37] or decrease [38,39] under warming conditions, regardless of the wet or dry climate background. By analysing previously reported data from 31 glaciers for 2012–2016 in High Mountain Asia [40], we found that the ablation gradients decreased significantly with increasing equilibrium line altitudes (ELAs) in both the relatively wet Indian monsoon and dry westerly climate domains (Fig. S5b). This spatial pattern is corroborated by temporal records: Interannual (6–8 years’ interval) to interdecadal (16–19 years’ interval) monitoring over the past 10–40 years has shown decreasing ablation gradients in several World Glacier Monitoring Service reference glaciers. These include Urumqi No. 1 [41,42] and Ts. Tuyuksuyskiy [42] in the westerly climate domain, as well as benchmark glaciers like Mera [43] and Yala [42] in the Indian monsoon climate domain (Fig. S5c–f). Together, these spatio-temporal observations, combined with our record of declining pollen radiocarbon ages from ~1860 to the 1990s, indicate regionally sustained decreasing ablation gradients across upstream glaciers feeding the seven lakes over the past 150 years (Fig. S5a and b). This likely drives a persistent upward expansion of the ablation zone, ensuring relatively greater meltwater input from young surface snow/ice than from old terminal ice, a process that may ultimately produce the observed decline in radiocarbon ages (Figs 2c and 3b).

However, in the third stage, after reaching their minimum values in the 1990s, the pollen radiocarbon ages abruptly increased over the last 30 years (Fig. 2c), indicating a dramatic input of meltwater from older ice into the glacier runoff. Under the relatively warm conditions of 2012–2016, ~60% of Tibetan glaciers retained >20% of their accumulation areas, providing a buffer that can be consumed as ablation zones expand under continued climate warming [40]. A stable or descending equilibrium line and increasing ablation gradients seem unlikely to occur (Fig. S5). Even within the western Kunlun, a core region of the Karakoram Anomaly [44], where glacier mass budgets remain balanced to slightly positive in contrast to widespread retreat elsewhere, the ELAs are indistinguishable from those of glaciers experiencing typical mass loss in adjacent regions [40]. This indicates that extra meltwater from old ice outside the enhanced surface melt system was fed into glacier runoff after the 1990s; otherwise, the pollen radiocarbon ages would show the same decreasing trends observed in the prior 130 years (i.e. a pattern of supraglacial melt). Therefore, the most likely cause of the abrupt increase in the pollen radiocarbon ages is the melting of old basal ice, triggered by the expansion and intensified drainage efficiency of subglacial hydrologic systems (Fig. 3c).

Three sources of basal heat, including geothermal flux, frictional heat and heat from surface melt input to the basal ice, can trigger this enhanced basal melting. Given the almost unchanged geothermal heat flux over the past century [45] and the overall 21st-century slowdown of Tibetan glaciers [46], we infer that stronger surface melt leads to an intensified input of surface meltwater to subglacial hydrologic systems, resulting in rising variations in the radiocarbon ages of pollen. The observed decreasing trends in the pollen radiocarbon ages in the prior 130 years indicate that the expanded upstream catchment of the ablation zone was large enough to counter the ‘aging’ effect of glacier terminus retreat on total glacier meltwater (Fig. 2c and Fig. S5). As climate warming and the upward expansion of the ablation zone continue, on the extensive crevassed surface in the downstream ablation zone, the enhanced melt condition and sufficient meltwater favour both hydrofracture [1,2,4,47,48] and vertical channel-incision [49] processes to route surface meltwater to the glacier bed. Through this proposed mechanism, both the down-glacier motion of the englacial passage and vertical channel incision reached the glacier bed in the 1990s, ultimately triggering increased old basal ice melt of the upstream glaciers in the seven lake catchments, thus reversing the trend of pollen radiocarbon ages thereafter (Figs 2c and 3).

Beyond climate warming, other factors—such as oscillations in the Indian monsoon and westerly precipitation and internal glacier dynamics—may also have contributed to the observed melt-regime shift. Enhanced post-LIA westerly precipitation [32], particularly during 1979–2010 [31,44], could have increased high-altitude snow accumulation and elevated driving stress, thereby favouring surge-like behaviour [15,44]. Glacier surges initiate when sustained mass build-up and rising basal water pressures suppress effective pressure and weaken basal traction, triggering rapid sliding and the formation of new crevasses [9,15,16,33]. Feedbacks among frictional heating, meltwater production and infiltration and high-pressure distributed drainage maintain fast flow until the subglacial hydrological system reorganizes into channelized conduits, lowering water pressure and restoring basal resistance [16,33]. The resulting hysteresis—driven by lags in thermal diffusion, progressive accumulation of subglacial water storage and drainage-system reconfiguration—helps govern decadal-to-centennial surge cycles [16,33]. Although the timing of Tibetan glacier surges remains uncertain, increased activity in the Karakoram after the 1990s [44] suggests that such cycles may overlap with our study interval. Meanwhile, climate warming has reduced monsoon snowfall and driven a snow-to-rain precipitation phase shift since 1975 [50]. This transition can enlarge the existing crevasses, deepen and prolong supraglacial stream incision and delay channel closure—conditions that favour the formation of englacial conduits capable of routing surface water to the glacier bed [1,2,4–6,49]. Whether these processes exerted influences comparable to or greater than climate warming remains uncertain, given limited constraints on long-term Tibetan glacier hydrology. Nevertheless, climate warming remains the most consistently supported and least controversial driver of post-LIA Tibetan glacier change [19,20,34,40,51].

In conclusion, our analyses of pollen radiocarbon ages from sediments in seven proglacial lakes suggest that sustained climate warming since the late 19th century [34] has likely triggered an abrupt shift in the melt regime of Tibetan glaciers. Before the 1990s, this regime was characterized by the persistent upward expansion of the ablation zone and intensification of young surface snow/ice melt, resulting in a continuous decrease in the pollen radiocarbon ages. After the 1990s, as indicated by the abrupt aging of the pollen ages, the melt regime was stamped by old basal ice melt driven by enhanced penetration of surface meltwater to the glacier bed under continued ablation zone expansion and stronger surface melt. On the glacier surface, accelerated thinning [51] and deepening incision of supraglacial streams [49] can establish a self-reinforcing feedback [49] that amplifies glacier disintegration (e.g. tongue separation and collapse) [52] and strengthens the coupling between supra- and subglacial hydrologic systems [1,2,4]. Through cryohydrologic warming and viscous dissipation, the meltwater flowing across and within the glacier can transfer heat to the glacier ice, further destabilizing the thermal regime and promoting basal ice melt [5,6,18,48]. These processes are likely to persist, accelerating the mass loss and disintegration of Tibetan glaciers. Model simulations project continued warming and potentially increasing precipitation on the Tibetan Plateau in the near future [53], implying the input of increased water (from snow and ice melt or even precipitation itself) into the basal ice of Tibetan glaciers. Current estimates of Tibetan glacier mass loss rely primarily on stake measurements of surface mass balance and satellite observations of total mass balance, which encompasses both surface and basal losses. Future assessments should account for supra–subglacial linkages to constrain evolving hydrothermal regimes and quantify the contribution of surface meltwater to internal and basal mass loss. Such investigations are critical because, unlike the Greenland Ice Sheet, Tibetan glaciers possess only a limited reservoir of cold content [54] and face a diminishing replenishment of cold ice [40].

## METHODS

Comprehensive descriptions of the climatic and geographic settings of the seven lake catchments, together with detailed sampling, dating, analytical procedures and interpretations are provided in Texts S1–S5.

We collected short sediment cores from seven proglacial lakes spanning two major climatic regimes on the Tibetan Plateau (Fig. 1). The site characteristics are summarized in Table S1. Everest Lake and Qiangyong Co, and the distal Tanggula Lake, lie within the semi-wet Indian monsoon domain, which received 380–480 mm of mean annual precipitation from 1979 to 2018. Four distal lakes—Aksai Chin, Guozha Co, Cangmu Co and Dong Co—are located in the semi-arid to arid westerly domain, where precipitation decreases from 300 mm at Dong Co to ~170–180 mm at Guozha Co and Aksai Chin. Mean annual temperatures vary by 9°C across the catchments, from −7.9°C in the western Kunlun to 1.5°C in south-western Qiangtang.

Catchment areas spanned ~1–6000 km², with upstream cold-based or polythermal glaciers covering 0.5%–55.0% of the basin area (Table S1). The glacier ELAs cluster between 5500 and 5900 m. Beyond glacierized zones, permafrost occupies most of the remaining terrain, with extents of 34.3%–88.1%, making cryospheric landforms the dominant landscape type (coverage >50% in six catchments). Vegetation cover, quantified using catchment-scale NDVI_max_, broadly followed the precipitation gradients: highest values occurred in the Tanggula Basin, whereas Aksai Chin and Guozha Co exhibited the lowest (Everest Lake is an exception due to its small catchment area).

To evaluate the hydrologic and sediment-supply sensitivity of glacier and permafrost processes, we calculated glacier and permafrost meltwater supply coefficients based on the physical basis of the lake supply coefficient. Everest Lake exhibited the highest values for both coefficients (63.0 and 474.5), one to two orders of magnitude greater than those of other sites, reflecting exceptionally strong glacier- and permafrost-driven sediment delivery. The influence of glacier processes declined eastward within the monsoon domain and more gradually across the westerlies, whereas the influence of permafrost processes exhibited no systematic spatial pattern (Fig. 2a and Table S1). The bedrock beneath all the upstream glaciers was broadly similar, consisting mainly of granite and metamorphic rocks, such as granitic gneiss, schists and slates (Table S1).

Sediment cores (24–50 cm) were retrieved in 2017–2018 using a UWITEC gravity corer at water depths of 2–83 m (Table S1). Most cores were sectioned at 1-cm intervals, except those from Everest Lake, Guozha Co and Aksai Chin (core 2, AKSQ‑2), which were sampled at 0.5-cm resolution.

Grain‑size distributions (0.01–3000 μm) for Qiangyong Co and both Aksai Chin cores were measured using a Mastersizer 3000 laser particle analyser following removal of organic matter (H₂O₂) and carbonates (HCl), and dispersion in sodium hexametaphosphate ((NaPO_3_)_6_).

The activities of ^210^Pb and ^137^Cs were measured using gamma spectrometry to establish the sediment chronologies. Age models were constructed using the R‑based *serac* package, applying piecewise CFCS, constant initial concentration (CIC) or constant rate of supply (CRS) models depending on the core characteristics (Software S1). Agreement with the 1955 and 1963 ^137^Cs markers (and 1986 where present) guided model selection, and the piecewise CFCS model was ultimately adopted for all lakes (Fig. S1a–g). Anomalous ^210^Pb_ex_ (unsupported or excess ^210^Pb) profiles in the upper 6 cm of Qiangyong Co were attributed to isotope dilution by coarse sandy clastic input (Fig. S2); the ages of these layers were estimated using the mean sedimentation rate of the underlying interval (Fig. S1b). For Aksai Chin, radionuclide data were available only for core 1 (AKSQ‑1) (Fig. S1d). The AKSQ‑2 chronology was derived by correlating sand‑content features between the two cores and applying a Bayesian age model using the *Bacon* package in R (Fig. S1h and i and Software S1).

Pollen grains were extracted from all sediment samples at temporal resolutions of 3–30 years. Each 10–20 g dry sample underwent acid–alkali–acid pretreatment (HCl, KOH, HF), followed by sieving at 125 μm to isolate macrofossils and 7 μm to eliminate clays. Heavy liquid separation (ZnI₂; densities 1.5, 1.8 and 2.1 g cm⁻³) was used to concentrate the pollen grains. Subsamples from five lakes were examined microscopically to confirm their overwhelmingly terrestrial origin and relatively good preservation quality (Figs S3 and S4). *Lycopodium* spores were added as markers, and ≥300 terrestrial pollen grains were counted where possible.

All concentrated pollen samples were submitted for AMS-^14^C dating at the Xi’an AMS Center. Of the 116 samples, 89 yielded measurable carbon, and ages were calibrated using CALIB 8.20 and IntCal20 (Dataset S1).

Because pollen grains in these lakes originate primarily from glacier meltwater, permafrost erosion and surface vegetation, we performed multiple linear regression analyses to quantify the relative contributions of these three sources to pollen radiocarbon age variations. Predictor variables included the glacier and permafrost meltwater supply coefficients and NDVI_max_. Corresponding ridge regressions were conducted to address the predictor collinearity. Analyses were performed in two climate domains for two temporal groups: post-2000 and the full period (Fig. S6 and Table S3). The post-2000 interval is emphasized because it reflects present-day conditions and enables space-for-time substitutions [24]. All regression analyses were conducted using the R-based *relaimpo, car* and *glmnet* packages.

Temporal trends in pollen radiocarbon ages were evaluated using linear models, Tukey tests, Davies’ tests and quadratic B‑splines in R software (Software S1). Most lakes exhibited significant nonlinear or piecewise trends, with breakpoints mainly clustered from the 1970s to the early 1990s (Table S4). The final B‑spline models indicated a broadly consistent shift from decreasing to increasing pollen radiocarbon ages around the 1990s across the seven lakes, suggesting a widespread change in the glacier melt regimes (Fig. 2c).

## Supplementary Material

nwag558_Supplemental_Files

## Data Availability

All sedimentary data generated from the seven proglacial lakes, together with the full reproducible code used for chronology and mathematical analyses, are provided in Dataset S1 and Software S1. Radiocarbon ages (derived from Δ^14^C) of soil organic matter from the 0–10 cm and 30–50 cm depth intervals across the Tibetan Plateau are available in reference [29] (Google Drive repository) and reference [28]. Meteorological parameters, glacier area, permafrost extent, NDVI_max_ and BCC-ESM1 historical NPP simulation (1850–2014) datasets are available in references listed in the Supplementary data.
