# Supplementary material for: Accelerated old basal ice loss in Tibetan glaciers after the 1990s
Source: Natl Sci Rev. 2026 Sep 4;13(19):nwag558. doi: 10.1093/nsr/nwag558 (PMC13752518; doi:10.1093/nsr/nwag558)
Supplement: Supplementary file 1 [file nwag558_supplemental_files.zip › Software_S1-Supplemantary_Data_ok.docx]

**Code utilised for chronology and mathematical analyses**

**For core chronology**

#evtools::install_github("rosalieb/serac",build_vignettes = TRUE)

library(serac)

vignette("serac")

setwd('data file ')

\# **1 for Everest Lake**

model_ZF <- serac(name="ZF", coring_yr=2018, model=c("CFCS","CIC"), plotphoto=FALSE, plot_Pb=T, plot_Am=F, plot_Cs=T, Cher = c(150,155), Hemisphere=c("NH"), NWT=c(190,195), FF=c(240,245), plotpdf=T, preview=T, save_code=F, mass_depth = F, sedchange = c(140))

\# **2 for Qiangyong Co**

model_QY <- serac(name="QY", coring_yr=2018, model=c("CFCS","CRS"), plotphoto=FALSE, plot_Pb=T, plot_Am=F, plot_Cs=T, Hemisphere=c("NH"), NWT=c(140,150), FF=c(190,200), plotpdf=T, preview=T, save_code=F, mass_depth = F, SML = 60,sedchange = c(160))

\# **3 for Tanggula Lake**

model_TGL <- serac(name="TGL", coring_yr=2017, model=c("CFCS","CIC"), plotphoto=FALSE, plot_Pb=T, plot_Am=F, plot_Cs=T, Hemisphere=c("NH"), NWT=c(110,120), FF=c(130,140), plotpdf=T, preview=T, save_code=F, mass_depth = F, sedchange =c(40,120))

\# **4 for core1 of Aksai Chin (AKSQ-1)**

model_AKSQ <- serac(name="AKSQ", coring_yr=2017, model=c("CFCS","CRS","CIC"), plotphoto=FALSE, plot_Pb=T, plot_Am=F, plot_Cs=T, Hemisphere=c("NH"), NWT=c(40,50), FF=c(50,60), plotpdf=T, preview=T, save_code=F, mass_depth = F, sedchange = c(90))

\# **5 for Guozha Co**

model_GZC <- serac(name="GZC", coring_yr=2017, model=c("CFCS","CRS","CIC"), plotphoto=FALSE, plot_Pb=T, plot_Am=F, plot_Cs=T, FF=c(40,50), Hemisphere=c("NH"), NWT=c(20,30), plotpdf=T, preview=T, save_code=F, mass_depth = F, sedchange = c(25))

\# **6 for Cangmu Co**

model_CMC <- serac(name="CMC", coring_yr=2017, model=c("CFCS","CRS","CIC"), plotphoto=FALSE, plot_Pb=T, plot_Am=F, plot_Cs=T, Hemisphere=c("NH"), NWT=c(40,50), plotpdf=T, preview=T, save_code=F, mass_depth = F, sedchange = c(25,130))

\# **7 for Dong Co**

model_DC <- serac(name="DC", coring_yr=2017, model=c("CFCS","CIC"), plotphoto=FALSE, plot_Pb=T, plot_Am=F, plot_Cs=T,Cher = c(60,70), Hemisphere=c("NH"), NWT=c(120,130), FF=c(150,160), plotpdf=T, preview=T, save_code=F, mass_depth = F, sedchange = c(160))

\# **8 for core2 of Aksai Chin (AKSQ-2)**

setwd('rbacon_3.3.1 file ')

library (rbacon)

Bacon(core="AKSQ2", cc=0, prob=0.95, postbomb=1, BCAD=TRUE)

Y

Y

**For trend analysis of pollen AMS-^14^C ages**

setwd('data file')

install.packages('pacman')

pacman::p_load(splines,ggplot2,cowplot,ggh4x,car,gvlma,caret,dplyr,tidyverse,segmented)

\# read data

QYC=read.csv("QYC.csv")

GZC=read.csv("GZC.csv")

TGL=read.csv("TGL.csv")

AKSQ=read.csv("AKSQ.csv")

CMC=read.csv("CMC.csv")

DC=read.csv("DC.csv")

ZF=read.csv("ZF.csv")

\# linear trend check for the data of each lake

\# **1-check for ZF (Everest Lake)**

ZF_line<-lm(X14C~yr,data=ZF)

summary(ZF_line)

residualPlots(ZF_line,fitted=T,smooth=T,quadratic=T) # note that 'smooth=T' displays result of a lowess smooth

\#	Test stat	Pr(>|Test stat|)

\# yr	3.5834	0.0698207

\# Tukey test	3.5834	0.0003392 ***

#Signif. codes: 0 ‘***’ 0.001 ‘**’ 0.01 ‘*’ 0.05 ‘.’ 0.1 ‘ ’ 1

\# **2-check for QYC (Qiangyong Co)**

QYC_line<-lm(X14C~yr,data=QYC)

summary(QYC_line)

residualPlots(QYC_line,fitted=T,smooth=T,quadratic=T)

\#	Test stat	Pr(>|Test stat|)

\# yr	2.7099	0.026663 *

\# Tukey test	2.7099	0.006731 **

\# Signif. codes: 0 ‘***’ 0.001 ‘**’ 0.01 ‘*’ 0.05 ‘.’ 0.1 ‘ ’ 1

\# **3-check for TGL (Tanggula Lake)**

TGL_line<-lm(X14C~yr,data=TGL)

summary(TGL_line)

residualPlots(TGL_line,fitted=T,smooth=T,quadratic=T)

\#	Test stat	Pr(>|Test stat|)

\# yr	2.2252	0.06771 .

\# Tukey test	2.2252	0.02607 *

\# Signif. codes: 0 ‘***’ 0.001 ‘**’ 0.01 ‘*’ 0.05 ‘.’ 0.1 ‘ ’ 1

\# **4-check for AKSQ (Aksai Chin)**

AKSQ_line<-lm(X14C~yr,data=AKSQ)

summary(AKSQ_line)

residualPlots(AKSQ_line,fitted=T,smooth=T,quadratic=T)

\#	Test stat Pr(>|Test stat|)

#yr	2.4925	0.02487 *

#Tukey test	2.4925	0.01268 *

#Signif. codes: 0 ‘***’ 0.001 ‘**’ 0.01 ‘*’ 0.05 ‘.’ 0.1 ‘ ’ 1

\# **5-check for GZC (Guozha Co)**

GZC_line<-lm(X14C~yr,data=GZC)

summary(GZC_line)

residualPlots(GZC_line,fitted=T,smooth=T,quadratic=T)

\#	Test stat	Pr(>|Test stat|)

\# yr	2.1605	0.07402 .

\# Tukey test	2.1605	0.03073 *

\# Signif. codes: 0 ‘***’ 0.001 ‘**’ 0.01 ‘*’ 0.05 ‘.’ 0.1 ‘ ’ 1

\# **6-check for CMC (Cangmu Co)**

CMC_line<-lm(X14C~yr,data=CMC)

summary(CMC_line)

residualPlots(CMC_line,fitted=T,smooth=T,quadratic=T)

\#	Test stat	Pr(>|Test stat|)

\# yr	1.0674	0.3068

\# Tukey test	1.0674	0.2858

\# further check for the linear trend of DC data

crPlots(CMC_line)

qqPlot(CMC_line)

\# test for normality in model residuals

shapiro.test(CMC_line$residuals)

\# Shapiro-Wilk normality test

\# data: CMC_line$residuals

\# W = 0.93769, p-value = 0.3542

\# test for non-constant variance in the model residuals

ncvTest(CMC_line)

\# Non-constant Variance Score Test

\# Variance formula: ~ fitted.values

\# Chisquare = 0.8721212, Df = 1, p = 0.35037

\# test for autocorrelation in the model residuals

durbinWatsonTest(CMC_line)

\# lag Autocorrelation D-W Statistic p-value

\# 1	0.1242564	1.568284	0.218

\# Alternative hypothesis: rho != 0

#add model to df

CMC<-cbind(CMC,predict(CMC_line,interval='confidence'))

#model performance

data.frame(RMSE=RMSE(CMC$fit,CMC$X14C),R2=R2(CMC$fit,CMC$X14C))

\#	RMSE	R2

\# 1 1082.252	0.1895309

\# **7-check for DC (Dong Co)**

DC_line<-lm(X14C~yr,data=DC)

summary(DC_line)

residualPlots(DC_line,fitted=T,smooth=T,quadratic=T)

\#	Test stat	Pr(>|Test stat|)

\# yr	1.5831	0.1299

\# Tukey test	1.5831	0.1134

\# further check for the linear trend of DC data

crPlots(DC_line)

qqPlot(DC_line)

\# test for normality in model residuals

shapiro.test(DC_line$residuals)

\# Shapiro-Wilk normality test

\# data: DC_line$residuals

\# W = 0.98499, p-value = 0.975

\# test for non-constant variance in the model residuals

ncvTest(DC_line)

\# Non-constant Variance Score Test

\# Variance formula: ~ fitted.values

\# Chisquare = 2.777008, Df = 1, p = 0.095627

\# test for autocorrelation in the model residuals

durbinWatsonTest(DC_line)

\# lag Autocorrelation D-W Statistic p-value

\# 1	0.3555695	1.254724	0.028

\# Alternative hypothesis: rho != 0

#add model to df

DC<-cbind(DC,predict(DC_line,interval='confidence'))

#model performance

data.frame(RMSE=RMSE(DC$fit,DC$X14C),R2=R2(DC$fit,DC$X14C))

\#	RMSE	R2

\# 1 740.7364	0.4549287

**Davies’ Test**

setwd("data file")

install.packages('pacman')

pacman::p_load(splines,ggplot2,cowplot,ggh4x,car,gvlma,caret,dplyr,tidyverse,segmented)

\# read data

QYC=read.csv("QYC.csv")

GZC=read.csv("GZC.csv")

TGL=read.csv("TGL.csv")

AKSQ=read.csv("AKSQ.csv")

CMC=read.csv("CMC.csv")

DC=read.csv("DC.csv")

ZF=read.csv("ZF.csv")

**#1 for ZF (Everest Lake)**

ZF_line<-lm(X14C~yr,data=ZF)

davies.test(ZF_line,~yr)

#Davies' test for a change in the slope

#data: formula = X14C ~ yr , method = lm

#model = gaussian , link = identity

#segmented variable = yr

#'best' at = 1979.3, n.points = 10, p-value = 0.2109

#alternative hypothesis: two.sided

**#2 for QYC (Qiangyong Co)**

QYC_line<-lm(X14C~yr,data=QYC)

davies.test(QYC_line,~yr)

#Davies' test for a change in the slope

#data: formula = X14C ~ yr , method = lm

#model = gaussian , link = identity

#segmented variable = yr

#'best' at = 1990.4, n.points = 10, p-value = 0.004832

#alternative hypothesis: two.sided

**#3 for TGL (Tanggula Lake)**

TGL_line<-lm(X14C~yr,data=TGL)

davies.test(TGL_line,~yr)

#Davies' test for a change in the slope

#data: formula = X14C ~ yr , method = lm

#model = gaussian , link = identity

#segmented variable = yr

#'best' at = 1981.7, n.points = 10, p-value = 0.1552

#alternative hypothesis: two.sided

**#4 for AKSQ (Aksai Chin)**

AKSQ_line<-lm(X14C~yr,data=AKSQ)

davies.test(AKSQ_line,~yr)

#Davies' test for a change in the slope

#data: formula = X14C ~ yr , method = lm

#model = gaussian , link = identity

#segmented variable = yr

#'best' at = 1986, n.points = 10, p-value = 0.004108

#alternative hypothesis: two.sided

**#5 for GZC (Guozha Co)**

GZC_line<-lm(X14C~yr,data=GZC)

davies.test(GZC_line,~yr)

#Davies' test for a change in the slope

#data: formula = X14C ~ yr , method = lm

#model = gaussian , link = identity

#segmented variable = yr

#'best' at = 1992.4, n.points = 10, p-value = 0.005443

#alternative hypothesis: two.sided

**#6 for CMC (Cangmu Co)**

CMC_line<-lm(X14C~yr,data=CMC)

davies.test(CMC_line,~yr)

#Davies' test for a change in the slope

#data: formula = X14C ~ yr , method = lm

#model = gaussian , link = identity

#segmented variable = yr

#'best' at = 1991, n.points = 10, p-value = 0.1926

#alternative hypothesis: two.sided

**#7 for DC (Dong Co)**

DC_line<-lm(X14C~yr,data=DC)

davies.test(DC_line,~yr)

#Davies' test for a change in the slope

#data: formula = X14C ~ yr , method = lm

#model = gaussian , link = identity

#segmented variable = yr

#'best' at = 1976.7, n.points = 10, p-value = 0.07757

#alternative hypothesis: two.sided

**Quadratic b-spline model for the data of each lake**

setwd('data file')

install.packages('pacman')

pacman::p_load(splines,ggplot2,cowplot,ggh4x,car,gvlma,caret,dplyr,tidyverse,segmented)

\# read data

QYC=read.csv("QYC.csv")

GZC=read.csv("GZC.csv")

TGL=read.csv("TGL.csv")

AKSQ=read.csv("AKSQ.csv")

CMC=read.csv("CMC.csv")

DC=read.csv("DC.csv")

ZF=read.csv("ZF.csv")

**#1 for ZF (Everest Lake)**

ZF$splines<-splines::bs(ZF$yr,degree=2,knots=c(1980))

ZF_spline<-lm(X14C ~ bs(yr,degree=2,knots = c(1980)),data=ZF)

summary(ZF_spline)

#Call:

#lm(formula = X14C ~ bs(yr, degree = 2, knots = c(1980)), data = ZF)

#Residuals:

\#	1	2	3	4	5

\# 164.209 -998.669 842.490 -14.937	6.907

#Coefficients:

\#	Estimate Std. Error t value Pr(>|t|)

#(Intercept)	17089	1317 12.976	0.049 *

#bs(yr, degree = 2, knots = c(1980))1	-7451	3831 -1.945	0.302

#bs(yr, degree = 2, knots = c(1980))2	-6673	2995 -2.228	0.269

#bs(yr, degree = 2, knots = c(1980))3	-1293	1856 -0.697	0.613

#Signif. codes: 0 ‘***’ 0.001 ‘**’ 0.01 ‘*’ 0.05 ‘.’ 0.1 ‘ ’ 1

#Residual standard error: 1317 on 1 degrees of freedom

#Multiple R-squared: 0.884,	Adjusted R-squared: 0.5359

#F-statistic: 2.539 on 3 and 1 DF, p-value: 0.4252

#add model to df

ZF<-cbind(ZF,predict(ZF_spline,interval='confidence'))

#model performance

data.frame(RMSE=RMSE(ZF$fit,ZF$X14C),R2=R2(ZF$fit,ZF$X14C))

\#	RMSE	R2

#1	588.9592	0.8839638

**#2 for QYC (Qiangyong Co)**

QYC$splines<-splines::bs(QYC$yr,degree=2,knots=c(1990))

QYC_spline<-lm(X14C ~ bs(yr,degree=2,knots = c(1990)),data=QYC)

summary(QYC_spline)

#Call:

#lm(formula = X14C ~ bs(yr, degree = 2, knots = c(1990)), data = QYC)

#Residuals:

\#	Min	1Q Median	3Q	Max

#-1695.2 -533.7	35.4	749.6 1534.1

#Coefficients:

\#	Estimate Std. Error t value Pr(>|t|)

#(Intercept)	9659.9	989.4	9.763 2.5e-05 ***

#bs(yr, degree = 2, knots = c(1990))1 -3953.6	2183.2 -1.811 0.11307

#bs(yr, degree = 2, knots = c(1990))2 -6534.9	1343.4 -4.865 0.00183 **

#bs(yr, degree = 2, knots = c(1990))3	1681.7	1604.4	1.048 0.32939

#Signif. codes: 0 ‘***’ 0.001 ‘**’ 0.01 ‘*’ 0.05 ‘.’ 0.1 ‘ ’ 1

#Residual standard error: 1257 on 7 degrees of freedom

#Multiple R-squared: 0.8411,	Adjusted R-squared: 0.7729

#F-statistic: 12.35 on 3 and 7 DF, p-value: 0.003488

\# check residudals

plot(QYC$yr,QYC_spline$residuals,xlab='yr',ylab='Residual')

abline(h=0)

plot(QYC_spline$fitted.values,QYC_spline$residuals,xlab='Fitted value',ylab='Residual')

abline(h=0)

crPlots(QYC_spline)

qqPlot(QYC_spline)

\# test for non-linearity

residualPlots(QYC_spline,fitted=T,smooth=T,quadratic=T)

\#	Test stat	Pr(>|Test stat|)

\# bs(yr, degree = 2, knots = c(1990))

\# Tukey test	0.3584	0.7201

\# test for normality in model residuals

shapiro.test(QYC_spline$residuals)

\# Shapiro-Wilk normality test

\# data: QYC_spline$residuals

\# W = 0.95665, p-value = 0.7293

\# test for non-constant variance in the model residuals

ncvTest(QYC_spline)

\# Non-constant Variance Score Test

\# Variance formula: ~ fitted.values

\# Chisquare = 0.3605483, Df = 1, p = 0.5482

\# test for autocorrelation in the model residuals

durbinWatsonTest(QYC_spline)

\#	lag Autocorrelation D-W Statistic p-value

\# 1	-0.4510679	2.689221	0.854

\# Alternative hypothesis: rho != 0

#add model to df

QYC<-cbind(QYC,predict(QYC_spline,interval='confidence'))

#model performance

data.frame(RMSE=RMSE(QYC$fit,QYC$X14C),R2=R2(QYC$fit,QYC$X14C))

\#	RMSE	R2

\# 1 1002.678	0.8410646

**#3 for TGL (Tanggula Lake)**

TGL$splines<-splines::bs(TGL$yr,degree=2,knots=c(1980))

TGL_spline<-lm(X14C ~ bs(yr,degree=2,knots = c(1980)),data=TGL)

summary(TGL_spline)

#Call:

#lm(formula = X14C ~ bs(yr, degree = 2, knots = c(1980)), data = TGL)

#Residuals:

\#	1	2	3	4	5	6	7	8	9

#-151.8 398.6 -249.9 -242.0 -156.4 543.1 -604.5 1208.7 -745.7

#Coefficients:

\#	Estimate Std. Error t value Pr(>|t|)

#(Intercept)	5006.7	622.9	8.038 0.000482 ***

#bs(yr, degree = 2, knots = c(1980))1 -1598.8	1371.5 -1.166 0.296317

#bs(yr, degree = 2, knots = c(1980))2 -3750.9	1109.2 -3.382 0.019641 *

#bs(yr, degree = 2, knots = c(1980))3 -1776.9	997.2 -1.782 0.134862

#Signif. codes: 0 ‘***’ 0.001 ‘**’ 0.01 ‘*’ 0.05 ‘.’ 0.1 ‘ ’ 1

#Residual standard error: 775.2 on 5 degrees of freedom

#Multiple R-squared: 0.7409,	Adjusted R-squared: 0.5854

#F-statistic: 4.766 on 3 and 5 DF, p-value: 0.06281

\# check residudals

plot(TGL$yr,TGL_spline$residuals,xlab='yr',ylab='Residual')

abline(h=0)

plot(TGL_spline$fitted.values,TGL_spline$residuals,xlab='Fitted value',ylab='Residual')

abline(h=0)

crPlots(TGL_spline)

qqPlot(TGL_spline)

\# test for non-linearity

residualPlots(TGL_spline,fitted=T,smooth=T,quadratic=T)

\#	Test stat	Pr(>|Test stat|)

\# bs(yr, degree = 2, knots = c(1980))

\# Tukey test	-0.9956	0.3195

\# test for normality in model residuals

shapiro.test(TGL_spline$residuals)

\# Shapiro-Wilk normality test

\# data: TGL_spline$residuals

\# W = 0.91676, p-value = 0.3661

\# test for non-constant variance in the model residuals

ncvTest(TGL_spline)

\# Non-constant Variance Score Test

\# Variance formula: ~ fitted.values

\# Chisquare = 3.048994, Df = 1, p = 0.080787

\# test for autocorrelation in the model residuals

durbinWatsonTest(TGL_spline)

\#	lag Autocorrelation	D-W Statistic	p-value

\# 1	-0.7012341	3.209734	0.376

\# Alternative hypothesis: rho != 0

#add model to df

TGL<-cbind(TGL,predict(TGL_spline,interval='confidence'))

#model performance

data.frame(RMSE=RMSE(TGL$fit,TGL$X14C),R2=R2(TGL$fit,TGL$X14C))

\#	RMSE	R2

\# 1 577.8097 0.7408968

**#4 for AKSQ (Aksai Chin)**

AKSQ$splines<-splines::bs(AKSQ$yr,degree=2,knots=c(1920,1970))

AKSQ_spline<-lm(X14C ~ bs(yr,degree=2,knots = c(1920,1970)),data=AKSQ)

summary(AKSQ_spline)

#Call:

#lm(formula = X14C ~ bs(yr, degree = 2, knots = c(1920, 1970)), data = AKSQ)

#Residuals:

\#	Min	1Q	Median	3Q	Max

#-1577.01 -549.77	0.68	377.33 3104.67

#Coefficients:

\#	Estimate Std. Error t value Pr(>|t|)

#(Intercept)	26741	1095 24.424 3.01e-12 ***

#bs(yr, degree = 2, knots = c(1920, 1970))1	-7238	1763 -4.105 0.001241 **

#bs(yr, degree = 2, knots = c(1920, 1970))2	-6321	1307 -4.835 0.000326 ***

#bs(yr, degree = 2, knots = c(1920, 1970))3	-14245	1811 -7.866 2.69e-06 ***

#bs(yr, degree = 2, knots = c(1920, 1970))4	-6755	1614 -4.185 0.001068 **

#Signif. codes: 0 ‘***’ 0.001 ‘**’ 0.01 ‘*’ 0.05 ‘.’ 0.1 ‘ ’ 1

#Residual standard error: 1228 on 13 degrees of freedom

#Multiple R-squared: 0.8656,	Adjusted R-squared: 0.8242

#F-statistic: 20.93 on 4 and 13 DF, p-value: 1.432e-05

\# check residudals

plot(AKSQ$yr,AKSQ_spline$residuals,xlab='yr',ylab='Residual')

abline(h=0)

plot(AKSQ_spline$fitted.values,AKSQ_spline$residuals,xlab='Fitted value',ylab='Residual')

abline(h=0)

crPlots(AKSQ_spline)

qqPlot(AKSQ_spline)

\# test for non-linearity

residualPlots(AKSQ_spline,fitted=T,smooth=T,quadratic=T)

\#	Test stat	Pr(>|Test stat|)

\# bs(yr, degree = 2, knots = c(1920,1970))

\# Tukey test	-0.9504	0.3419

\# test for normality in model residuals

shapiro.test(AKSQ_spline$residuals)

\# Shapiro-Wilk normality test

\# data: AKSQ_spline$residuals

\# W = 0.90054, p-value = 0.05871

\# test for non-constant variance in the model residuals

ncvTest(AKSQ_spline)

\# Non-constant Variance Score Test

\# Variance formula: ~ fitted.values

\# Chisquare = 0.9847594, Df = 1, p = 0.32103

\# test for autocorrelation in the model residuals

durbinWatsonTest(AKSQ_spline)

\#	lag Autocorrelation	D-W Statistic	p-value

\# 1	-0.4247044	2.82876	0.332

\# Alternative hypothesis: rho != 0

#add model to df

AKSQ<-cbind(AKSQ,predict(AKSQ_spline,interval='confidence'))

#model performance

data.frame(RMSE=RMSE(AKSQ$fit,AKSQ$X14C),R2=R2(AKSQ$fit,AKSQ$X14C))

\#	RMSE	R2

#1 1043.802 0.8655981

**#5 for GZC (Guozha Co)**

GZC$splines<-splines::bs(GZC$yr,degree=2,knots=c(1980))

GZC_spline<-lm(X14C ~ bs(yr,degree=2,knots = c(1980)),data=GZC)

summary(GZC_spline)

#Call:

#lm(formula = X14C ~ bs(yr, degree = 2, knots = c(1980)), data = GZC)

#Residuals:

\#	1	2	3	4	5	6	7	8	9

#-1221.93 1864.25 -561.05	-10.27 -797.30	446.32 1001.65 -1101.90	380.23

#Coefficients:

\#	Estimate Std. Error t value Pr(>|t|)

#(Intercept)	19974	1075 18.576 8.32e-06 ***

#bs(yr, degree = 2, knots = c(1980))1	-1217	2508 -0.485 0.64801

#bs(yr, degree = 2, knots = c(1980))2	-11397	1678 -6.790 0.00105 **

#bs(yr, degree = 2, knots = c(1980))3	1672	1586	1.055 0.33990

#Signif. codes: 0 ‘***’ 0.001 ‘**’ 0.01 ‘*’ 0.05 ‘.’ 0.1 ‘ ’ 1

#Residual standard error: 1302 on 5 degrees of freedom

#Multiple R-squared: 0.922,	Adjusted R-squared: 0.8752

#F-statistic: 19.7 on 3 and 5 DF, p-value: 0.003363

\# check residudals

plot(GZC$yr,GZC_spline$residuals,xlab='yr',ylab='Residual')

abline(h=0)

plot(GZC_spline$fitted.values,GZC_spline$residuals,xlab='Fitted value',ylab='Residual')

abline(h=0)

crPlots(GZC_spline)

qqPlot(GZC_spline)

\# test for non-linearity

residualPlots(GZC_spline,fitted=T,smooth=T,quadratic=T)

\#	Test stat	Pr(>|Test stat|)

\# bs(yr, degree = 2, knots = c(1980))

\# Tukey test	-2.2174	0.0266 *

\# Signif. codes: 0 ‘***’ 0.001 ‘**’ 0.01 ‘*’ 0.05 ‘.’ 0.1 ‘ ’ 1

\# test for normality in model residuals

shapiro.test(GZC_spline$residuals)

\# Shapiro-Wilk normality test

\# data: GZC_spline$residuals

\# W = 0.94764, p-value = 0.6641

\# test for non-constant variance in the model residuals

ncvTest(GZC_spline)

\# Non-constant Variance Score Test

\# Variance formula: ~ fitted.values

\# Chisquare = 0.541242, Df = 1, p = 0.46192

\# test for autocorrelation in the model residuals

durbinWatsonTest(GZC_spline)

\#	lag Autocorrelation	D-W Statistic	p-value

\# 1	-0.5591106	2.925106	0.888

\# Alternative hypothesis: rho != 0

#add model to df

GZC<-cbind(GZC,predict(GZC_spline,interval='confidence'))

#model performance

data.frame(RMSE=RMSE(GZC$fit,GZC$X14C),R2=R2(GZC$fit,GZC$X14C))

\#	RMSE	R2

#1 970.7012 0.9220059

\# **6 for CMC (Cangmu Co)**

CMC$splines<-splines::bs(CMC$yr,degree=2,knots=c(1950))

CMC_spline<-lm(X14C ~ bs(yr,degree=2,knots = c(1950)),data=CMC)

summary(CMC_spline)

#Call:

#lm(formula = X14C ~ bs(yr, degree = 2, knots = c(1950)), data = CMC)

#Residuals:

\#	Min	1Q	Median	3Q	Max

#-1447.88 -395.98	-3.61	518.62 1496.14

#Coefficients:

\#	Estimate Std. Error t value Pr(>|t|)

#(Intercept)	8570.61	811.40 10.563 4.27e-07 ***

#bs(yr, degree = 2, knots = c(1950))1	935.79	1424.90	0.657 0.52485

#bs(yr, degree = 2, knots = c(1950))2 -3464.56	1066.19 -3.249 0.00774 **

#bs(yr, degree = 2, knots = c(1950))3	-89.87	1234.68 -0.073 0.94328

#Signif. codes: 0 ‘***’ 0.001 ‘**’ 0.01 ‘*’ 0.05 ‘.’ 0.1 ‘ ’ 1

#Residual standard error: 978 on 11 degrees of freedom

#Multiple R-squared: 0.5147,	Adjusted R-squared: 0.3823

#F-statistic: 3.888 on 3 and 11 DF, p-value: 0.04059

\# check residudals

plot(CMC$yr,CMC_spline$residuals,xlab='yr',ylab='Residual')

abline(h=0)

plot(CMC_spline$fitted.values,CMC_spline$residuals,xlab='Fitted value',ylab='Residual')

abline(h=0)

crPlots(CMC_spline)

qqPlot(CMC_spline)

\# test for non-linearity

residualPlots(CMC_spline,fitted=T,smooth=T,quadratic=T)

\#	Test stat	Pr(>|Test stat|)

\# bs(yr, degree = 2, knots = c(1950))

\# Tukey test	0.8326	0.4051

\# test for normality in model residuals

shapiro.test(CMC_spline$residuals)

\# Shapiro-Wilk normality test

\# data: CMC_spline$residuals

\# W = 0.9686, p-value = 0.8368

\# test for non-constant variance in the model residuals

ncvTest(CMC_spline)

\# Non-constant Variance Score Test

\# Variance formula: ~ fitted.values

\# Chisquare = 0.7415859, Df = 1, p = 0.38915

\# test for autocorrelation in the model residuals

durbinWatsonTest(CMC_spline)

\#	lag Autocorrelation D-W Statistic	p-value

\#	1	-0.1293556	2.2587	0.69

\# Alternative hypothesis: rho != 0

#add model to df

CMC<-cbind(CMC,predict(CMC_spline,interval='confidence'))

#model performance

data.frame(RMSE=RMSE(CMC$fit,CMC$X14C),R2=R2(CMC$fit,CMC$X14C))

\#	RMSE	R2

\# 1 837.4951 0.5146621

**#7 for DC (Dong Co)**

DC$splines<-splines::bs(DC$yr,degree=2,knots=c(1975))

DC_spline<-lm(X14C ~ bs(yr,degree=2,knots = c(1975)),data=DC)

summary(DC_spline)

#Call:

#lm(formula = X14C ~ bs(yr, degree = 2, knots = c(1975)), data = DC)

#Residuals:

\#	Min	1Q Median	3Q	Max

#-1216.3 -457.5 -120.1	361.8 1262.8

#Coefficients:

\#	Estimate Std. Error t value Pr(>|t|)

#(Intercept)	8074.0	479.4 16.843 1.83e-12 ***

#bs(yr, degree = 2, knots = c(1975))1 -1192.5	983.1 -1.213 0.240818

#bs(yr, degree = 2, knots = c(1975))2 -2629.4	563.2 -4.669 0.000191 ***

#bs(yr, degree = 2, knots = c(1975))3 -1359.9	805.0 -1.689 0.108408

#Signif. codes: 0 ‘***’ 0.001 ‘**’ 0.01 ‘*’ 0.05 ‘.’ 0.1 ‘ ’ 1

#Residual standard error: 733.7 on 18 degrees of freedom

#Multiple R-squared: 0.5625,	Adjusted R-squared: 0.4896

#F-statistic: 7.715 on 3 and 18 DF, p-value: 0.00161

\# check residudals

plot(DC$yr,DC_spline$residuals,xlab='yr',ylab='Residual')

abline(h=0)

plot(DC_spline$fitted.values,DC_spline$residuals,xlab='Fitted value',ylab='Residual')

abline(h=0)

crPlots(DC_spline)

qqPlot(DC_spline)

\# test for non-linearity

residualPlots(DC_spline,fitted=T,smooth=T,quadratic=T)

\# bs(yr, degree = 2, knots = c(1975))

\# Tukey test	-1.6473	0.09949 .

\# Signif. codes: 0 ‘***’ 0.001 ‘**’ 0.01 ‘*’ 0.05 ‘.’ 0.1 ‘ ’ 1

\# test for normality in model residuals

shapiro.test(DC_spline$residuals)

\# Shapiro-Wilk normality test

\# data: DC_spline$residuals

\# W = 0.96137, p-value = 0.5174

\# test for non-constant variance in the model residuals

ncvTest(DC_spline)

\# Non-constant Variance Score Test

\# Variance formula: ~ fitted.values

\# Chisquare = 2.696817, Df = 1, p = 0.10055

\# test for autocorrelation in the model residuals

durbinWatsonTest(DC_spline)

\#	lag Autocorrelation D-W Statistic	p-value

\#	1	0.2262267	1.523135	0.058

\#	Alternative hypothesis: rho != 0

#add model to df

DC<-cbind(DC,predict(DC_spline,interval='confidence'))

#model performance

data.frame(RMSE=RMSE(DC$fit,DC$X14C),R2=R2(DC$fit,DC$X14C))

\#	RMSE	R2

#1 663.6149 0.56252

#plot graphs

p1=ggplot(ZF,aes(yr,X14C))+

geom_point(shape=21,size=3,stroke=0.5,color="white",fill="#009ACD",alpha=0.2)+

geom_errorbar(aes(xmin=yr-yrsd,xmax=yr+yrsd),width=0,col='lightgrey')+

geom_errorbar(aes(ymin=X14C-X14Closd,ymax=X14C+X14Cupsd),width=0,col='lightgrey')+

labs(x=NULL,y=y1<-expression("Pollen AMS-"^14*"C"~age~(cal~yr~BP)))+

theme_bw()+

theme(panel.grid=element_blank())+

scale_x_continuous(limits=c(1860,2020),

breaks=seq(1860,2020,20),

guide="axis_minor",

minor_breaks=seq(1870,2010,20))+

guides(x=guide_axis_minor(trunc_lower = 1860,trunc_upper = 2020))+

scale_y_continuous(position="right",

limits=c(9000,19000),

breaks=seq(10000,18000,4000),

minor_breaks = seq(9000,19000,1000))+

stat_smooth(method=lm,formula=y~splines::bs(x,degree=2,knots=c(1980)),lwd=1,color="red",fill="#FFB6C1",alpha=0.15)+

coord_flip()

p2=ggplot(QYC,aes(yr,X14C))+

geom_point(shape=21,size=3,stroke=0.5,color="white",fill="#009ACD",alpha=0.2)+

geom_errorbar(aes(xmin=yr-yrsd,xmax=yr+yrsd),width=0,col='lightgrey')+

geom_errorbar(aes(ymin=X14C-X14Closd,ymax=X14C+X14Cupsd),width=0,col='lightgrey')+

labs(x=NULL,

y=y1<-expression("Pollen AMS-"^14*"C"~age~(cal~yr~BP)))+

theme_bw()+

theme(panel.grid=element_blank())+

scale_x_continuous(limits=c(1860,2020),

breaks=seq(1860,2020,20),

guide="axis_minor",

minor_breaks=seq(1870,2010,20))+

guides(x=guide_axis_minor(trunc_lower = 1860,trunc_upper = 2020))+

scale_y_continuous(position="right",

limits=c(500,15000),

breaks=seq(1000,13000,6000),

minor_breaks = seq(2000,15000,1000))+

stat_smooth(method=lm,formula=y~splines::bs(x,degree=2,knots=c(1990)),lwd=1,color="red",fill="#FFB6C1",alpha=0.15)+

coord_flip()+

theme(axis.text.y=element_blank(),

axis.ticks.y =element_blank())

p3=ggplot(TGL,aes(yr,X14C))+

geom_point(shape=21,size=3,stroke=0.5,color="white",fill="#009ACD",alpha=0.2)+

geom_errorbar(aes(xmin=yr-yrsd,xmax=yr+yrsd),width=0,col='lightgrey')+

geom_errorbar(aes(ymin=X14C-X14Closd,ymax=X14C+X14Cupsd),width=0,col='lightgrey')+

labs(x=NULL,y=y1<-expression("Pollen AMS-"^14*"C"~age~(cal~yr~BP)))+

theme_bw()+

theme(panel.grid=element_blank())+

scale_x_continuous(limits=c(1860,2020),

breaks=seq(1860,2020,20),

guide="axis_minor",

minor_breaks=seq(1870,2010,20))+

guides(x=guide_axis_minor(trunc_lower = 1860,trunc_upper = 2020))+

scale_y_continuous(position="right",

limits=c(-200,8000),

breaks=seq(1000,7000,3000),

minor_breaks = seq(2000,6000,1000))+

stat_smooth(method=lm,formula=y~splines::bs(x,degree=2,knots=c(1980)),lwd=1,color="red",fill="#FFB6C1",alpha=0.15)+

coord_flip()+

theme(axis.text.y=element_blank(),

axis.ticks.y =element_blank())

p4=ggplot(AKSQ,aes(yr,X14C))+

geom_point(shape=21,size=3,stroke=0.5,color="white",fill="#009ACD",alpha=0.2)+

geom_errorbar(aes(xmin=yr-yrsd,xmax=yr+yrsd),width=0,col='lightgrey')+

geom_errorbar(aes(ymin=X14C-X14Closd,ymax=X14C+X14Cupsd),width=0,col='lightgrey')+

labs(x=NULL,y=y1<-expression("Pollen AMS-"^14*"C"~age~(cal~yr~BP)))+

theme_bw()+

theme(panel.grid=element_blank())+

scale_x_continuous(limits=c(1860,2020),

breaks=seq(1860,2020,20),

guide="axis_minor",

minor_breaks=seq(1870,2010,20))+

guides(x=guide_axis_minor(trunc_lower = 1860,trunc_upper = 2020))+

scale_y_continuous(position="right",

limits=c(9000,32000),

breaks=seq(12000,28000,8000),

minor_breaks = seq(10000,30000,1000))+

stat_smooth(method=lm,formula=y~splines::bs(x,degree=2,knots=c(1920,1970)),lwd=1,color="red",fill="#FFB6C1",alpha=0.15)+

coord_flip()+

theme(axis.text.y=element_blank(),

axis.ticks.y =element_blank())

p5=ggplot(GZC,aes(yr,X14C))+

geom_point(shape=21,size=3,stroke=0.5,color="white",fill="#009ACD",alpha=0.2)+

geom_errorbar(aes(xmin=yr-yrsd,xmax=yr+yrsd),width=0,col='lightgrey')+

geom_errorbar(aes(ymin=X14C-X14Closd,ymax=X14C+X14Cupsd),width=0,col='lightgrey')+

labs(x=NULL,y=y1<-expression("Pollen AMS-"^14*"C"~age~(cal~yr~BP)))+

theme_bw()+

theme(panel.grid=element_blank())+

scale_x_continuous(limits=c(1860,2020),

breaks=seq(1860,2020,20),

guide="axis_minor",

minor_breaks=seq(1870,2010,20))+

guides(x=guide_axis_minor(trunc_lower = 1860,trunc_upper = 2020))+

scale_y_continuous(position="right",

limits=c(6000,25000),

breaks=seq(9000,23000,7000),

minor_breaks = seq(8000,24000,1000))+

stat_smooth(method=lm,formula=y~splines::bs(x,degree=2,knots=c(1980)),lwd=1,color="red",fill="#FFB6C1",alpha=0.15)+

coord_flip()+

theme(axis.text.y=element_blank(),

axis.ticks.y =element_blank())

p6=ggplot(CMC,aes(yr,X14C))+

geom_point(shape=21,size=3,stroke=0.5,color="white",fill="#009ACD",alpha=0.2)+

geom_errorbar(aes(xmin=yr-yrsd,xmax=yr+yrsd),width=0,col='lightgrey')+

geom_errorbar(aes(ymin=X14C-X14Closd,ymax=X14C+X14Cupsd),width=0,col='lightgrey')+

labs(x=NULL,y=y1<-expression("Pollen AMS-"^14*"C"~age~(cal~yr~BP)))+

theme_bw()+

theme(panel.grid=element_blank())+

scale_x_continuous(limits=c(1860,2020),

breaks=seq(1860,2020,20),

guide="axis_minor",

minor_breaks=seq(1870,2010,20))+

guides(x=guide_axis_minor(trunc_lower = 1860,trunc_upper = 2020))+

scale_y_continuous(position="right",

limits=c(4000,11000),

breaks=seq(5000,11000,3000),

minor_breaks = seq(3000,12000,1000))+

stat_smooth(method=lm,formula=y~splines::bs(x,degree=2,knots=c(1950)),lwd=1,color="red",fill="#FFB6C1",alpha=0.15)+

coord_flip()+

theme(axis.text.y=element_blank(),

axis.ticks.y =element_blank())

p7=ggplot(DC,aes(yr,X14C))+

geom_point(shape=21,size=3,stroke=0.5,color="white",fill="#009ACD",alpha=0.2)+

geom_errorbar(aes(xmin=yr-yrsd,xmax=yr+yrsd),width=0,col='lightgrey')+

geom_errorbar(aes(ymin=X14C-X14Closd,ymax=X14C+X14Cupsd),width=0,col='lightgrey')+

labs(x=NULL,y=y1<-expression("Pollen AMS-"^14*"C"~age~(cal~yr~BP)))+

theme_bw()+

theme(panel.grid=element_blank())+

scale_x_continuous(limits=c(1860,2020),

breaks=seq(1860,2020,20),

guide="axis_minor",

minor_breaks=seq(1870,2010,20))+

guides(x=guide_axis_minor(trunc_lower = 1860,trunc_upper = 2020))+

scale_y_continuous(position="right",

limits=c(3000,11000),

breaks=seq(4000,10000,3000),

minor_breaks = seq(5000,9000,1000))+

stat_smooth(method=lm,formula=y~splines::bs(x,degree=2,knots=c(1975)),lwd=1,color="red",fill="#FFB6C1",alpha=0.15)+

coord_flip()+

theme(axis.text.y=element_blank(),

axis.ticks.y =element_blank())

plot_grid(p1,p2,p3,p4,p5,p6,p7,nrow=1,ncol=7,align="v",rel_widths = c(2,2,2,2,2,2,2))

**Multiple linear and ridge regression analyses**

library(relaimpo)

library(car)

library(glmnet)

\# read data

Westerly_Post2000 <- read.csv("datafile/WesterlyPost2000.csv")

\# 1. Standardize all variables

Westerly_Post2000scaled <- as.data.frame(scale(Westerly_Post2000))

\# 2. Multiple linear analysis on scaled data

Westerly_Post2000model <- lm(Radiocarbon ~ Permafrost + Glacier + NDVImax, data = Westerly_Post2000scaled)

summary(Westerly_Post2000model)

#Diagnosis

#par(mfrow=c(2,2))

#plot(Westerly_Post2000model)

#3. Multiple linear analysis on original data

Westerly_Post2000modeloriginal <- lm(Radiocarbon ~ Permafrost + Glacier + NDVImax, data = Westerly_Post2000)

#calculate relative importance

rel <- calc.relimp(Westerly_Post2000modeloriginal, type = "lmg")

rel

\# calculate VIF

vif(Westerly_Post2000modeloriginal)

avPlots(Westerly_Post2000modeloriginal, ask=FALSE)

#Owing to collinearity, ridge regression was applied

\# 1. Standardize variables

X <- scale(as.matrix(Westerly_Post2000 [, c("Permafrost", "Glacier"," NDVImax")]))

Y <- scale(Westerly_Post2000$Radiocarbon)

\# 2. LOOCV (n=26)

set.seed(123)

cv_ridge <- cv.glmnet(X, Y, alpha = 0, nfolds = 26)

\# 3. Best λ

best_lambda <- cv_ridge$lambda.min

best_lambda

\# 4. Coefficients under best lambda

ridge_coef <- coef(cv_ridge, s = "lambda.min")

ridge_coef

\# 5. Ridge path

plot(glmnet(X, Y, alpha = 0), xvar = "lambda", label = TRUE)

abline(v = log(best_lambda), col = "red", lwd = 2)

\# 6. CV curve

plot(cv_ridge)

\# **The same code can be applied to both the full‑period dataset and the three monsoon‑domain lake datasets, with the dataset names and the value of nfolds needing adjustment.**
