# Supplementary material for: Accelerated old basal ice loss in Tibetan glaciers after the 1990s
Source: Natl Sci Rev. 2026 Sep 4;13(19):nwag558. doi: 10.1093/nsr/nwag558 (PMC13752518; doi:10.1093/nsr/nwag558)
Supplement: Supplementary file 1 [file nwag558_supplemental_files.zip › Supplementary_Data-Clean_version_ok.docx]

Supplementary Data to:

**Accelerated old basal ice loss in Tibetan glaciers after the 1990s**

Baiqing Xu^1,#,*^, Xiaolong Zhang^1,#^, Tandong Yao^1,*^, Wei Yang^1^, Xiangke Xu^1^

^1^ State Key Laboratory of Tibetan Plateau Earth System, Environment and Resources (TPESER), Institute of Tibetan Plateau Research, Chinese Academy of Sciences; Beijing, 100101, China

^#^ These authors contributed equally: Baiqing Xu, Xiaolong Zhang

* Corresponding author: Baiqing Xu, Tandong Yao; Email: baiqing@itpcas.ac.cn, tdyao@itpcas.ac.cn

**This file includes:**

Text S1 to S5

Figure S1 to S8

Table S1 to S4

SI References

Legend for Dataset S1

Legend for Software S1

**Other supporting materials for this manuscript include the following:**

Dataset S1 (separate table file)

Software S1 (separate text file)

**Supplementary Data Text**

**Text S1: Climatic, geographic, and geologic settings**

We sampled short sediment cores from seven proglacial lakes in two climate domains on the Tibetan Plateau (Fig. 1). Detailed climatic, geographic, and geologic information, together with precise core locations, is listed in Table S1. Among these lakes, Everest Lake and Qiangyong Co are proximal proglacial lakes located in the central-eastern Himalayas. Tanggula Lake is a distal proglacial lake located in the central Tanggula Mountains. These three catchments lie within the semi-wet Indian monsoon domain [22,31]. According to the China meteorological forcing dataset [55], the mean annual precipitation from1979 to 2018 was 380 mm at Qiangyong Co and approximately 480 mm at Everest Lake and Tanggula Lake. Aksai Chin, Guozha Co, Cangmu Co, and Dong Co are distal proglacial lakes located within semi-arid to arid westerly domain [22,31]. Aksai Chin and Guozha Co lie in the western Kunlun, a classical westerly-dominated region, whereas Cangmu Co and Dong Co occupy the southwestern Qiangtang Plateau near the boundary between the westerlies and Indian monsoon [22]. The mean annual precipitation (1979–2018) [55] decreased from 300 mm at Dong Co to approximately 170–180 mm at Guozha Co and Aksai Chin. The mean annual temperature (1979–2018) [55] varied by 9 ℃ across the seven catchments, from –7.9 and –6.0 ℃ in the western Kunlun to –0.1 and 1.5 ℃ in the southwestern Qiangtang.

The catchment areas spanned nearly three orders of magnitude (1–6000 km^2^). Upstream glaciers, primarily cold-based and polythermal [14], cover 0.5–55.0% of the basin area [56–59] (Table S1). The current regional equilibrium line altitudes of these glaciers ranged from approximately 5500 to 5900 m [40,59,60] (Table S1). The permafrost extent was calculated by summing the continuous, discontinuous, sporadic, and isolated permafrost classes [61], yielding a basin-scale coverage of 34.3–88.1% (highest in the Everest Basin and lowest in the Dong Co Basin; Table S1). When combined with glacierised areas, cryospheric landforms occupied > 50% of the six catchments (51.2–99.8%), with Dong Co being the only exception (34.8%).

Vegetation development was characterised using the basin-scale median annual maximum Normalised Difference Vegetation Index (NDVI_max_) derived from reference [62]. NDVI_max_ broadly follows precipitation gradients [63], with dense vegetation in the wet Tanggula basin (0.356) and sparse cover in the dry Aksai Chin (0.088) and Guozha Co (0.087) basins. Everest Lake is an exception because of its small catchment area relative to the 1-km spatial resolution of the NDVI_max_ dataset.

To assess the sensitivity of sediment delivery to cryospheric processes, we calculated glacier and permafrost meltwater supply coefficients following the physical basis of the lake supply coefficient, which relates catchment-to-lake area ratio to the influence of upstream runoff on lake water and sediment balance [64]. Everest Lake exhibited the highest values of both coefficients—one to two orders of magnitude greater than the other lakes (63.0 and 474.5 versus 0.3–8.3 and 5.8–26.2; Fig. 2a and Table S1)—indicating exceptionally strong glacier- and permafrost-driven sediment input. In the Indian monsoon climate domain, the glacier meltwater supply coefficient decreased markedly from 63.0 in Everest Lake to 8.3 in Qiangyong Co and 2.0 in Tanggula Lake catchments. In the westerly climate domain, the glacier meltwater supply coefficient decreased more gradually, with values of 2.6, 2.2, 0.9, and 0.3 in the Aksai Chin, Guozha Co, Cangmu Co, and Dong Co catchments, respectively. In contrast, the permafrost meltwater supply coefficient showed no systematic spatial patterns, with the highest value in the Everest Basin and the lowest value in Qiangyong Co.

Despite spanning a broad range of climatic and glacier regimes, the bedrock lithologies beneath the upstream glaciers of the seven lakes were relatively uniform, primarily composed of granite and metamorphic rocks such as granitic gneiss, schists, and slates [65–70] (Table S1).

**Text S2: Sediment coring and analysis**

Sediment cores, ranging from 24 to 50 cm in length, were recovered from the seven lakes using a UWITEC gravity corer (UWITEC GmbH, Austria) at water depths of 2–83 m in 2017 or 2018 (Table S1). Except for two cores in Aksai Chin (AKSQ-1 and AKSQ-2), one sediment core was sampled from each lake. The sediment cores from Everest Lake, Guozha Co, and Aksai Chin (AKSQ-2) were sliced at 0.5 cm intervals, whereas the others were sliced at 1 cm intervals. All sliced sediment samples were freeze-dried and analysed at the Key Laboratory of Tibetan Plateau Earth System, Environment and Resources, Institute of Tibetan Plateau Research.

The activities of ^210^Pb and ^137^Cs in the entire sediment samples of Everest Lake, Qiangyong Co, Tanggula Lake, and Dong Co, as well as in the top 12 cm of sediment samples from AKSQ-1, the top 10 cm from Guozha Co (with adjacent samples pooled and measured at 1 cm intervals), and the top 20 cm from Cangmu Co, were measured using gamma spectrometry with a well-type Ge detector (Ortec HPGe GWL, USA). Specifically, total ^210^Pb and ^137^Cs were determined from their gamma emissions at 46.5 keV and 662 keV, respectively. ^226^Ra was determined from the gamma emissions of its daughter isotope ^214^Pb at 295.2 and 352 keV. Excess (Unsupported) ^210^Pb (^210^Pb_ex_) was calculated by subtracting ^226^Ra from the total ^210^Pb. Standard radioactive sources of ^210^Pb, ^226^Ra, and ^137^Cs, produced by the China Institute of Atomic Energy, were used to determine the efficiency of the spectrometer. The uncertainties in the original activities were 5.0% for ^210^Pb, 4.5% for ^226^Ra, and 3.6% for ^137^Cs, respectively.

After establishing the chronologies of all sediment cores, pollen grains were extracted from all lake sediment samples at time resolutions ranging from 3 to 30 years (with some sediment samples pooled) (Dataset S1), considering the sediment volume of each sample and the previously reported very low content of sedimentary organic matter in Tibetan lakes [71]. For pollen extraction, sediment samples with a dry weight of 10–20 g were treated with acid-alkali-acid procedures using 36% HCl, 10% KOH, and 40% HF. Before soaking in HF, the samples were sieved with a 125 μm screen to identify and select plant macrofossil fragments. After treatment with HF, the samples were sieved with a 7 μm screen to remove clay-sized particles, and pollen grains on the sieve were collected. The extracted pollen grains were further purified and concentrated by stepwise heavy liquid (ZnI_2_) density separation at three different densities (1.5, 1.8, and 2.1 g cm^-3^). To confirm that the pollen grains were predominantly derived from terrestrial vegetation and not contaminated by non-terrestrial organic matter, such as lake algae, for further radiocarbon age analysis, 3 samples from Guozha Co, 6 from Everest Lake, 6 from AKSQ-1, 5 from Cangmu Co, and 5 from Dong Co were picked and split into two parts with volumes of 100 µl and 400 µl, respectively. The 100 µl samples were then sent to the Hebei Key Laboratory of Environmental Change and Ecological Construction, Hebei Normal University, for pollen identification under a Leica DM5500 B microscope, using *The Pollen Flora of China* [72] and *Atlas of Quaternary Pollen and Spores in China* [73] as references. *Lycopodium* spores (10315 grains) were added before microscopic analysis, and more than 300 terrestrial pollen grains were counted in each sample. However, due to low concentrations, 200–300 terrestrial pollen grains were counted in 3 samples, and fewer than 200 in 5 samples (Fig. S3 and Dataset S1). Pollen percentages were calculated based on the total number of pollen grains, and pollen diagrams were plotted using Tilia software [74]. Microscopic analysis revealed relatively fresh pollen grains (Fig. S4), suggesting good preservation and similarity to pollen components preserved in a recently reported Tibetan ice core [75]. Aquatic pollen grains represent only 0–0.95% (0–3 grains) of the total counted assemblages (69–479 grains; Dataset S1), confirming that pollen in our lake sediments is overwhelmingly terrestrial in origin (Fig. S3). This pattern aligns with a recent synthesis of pollen data from 90 lakes across the Tibetan Plateau [76], which reported dominant terrestrial inputs (~95%) and a strong contribution from herbaceous taxa (~76%) in surface sediments. Because aquatic plants may incorporate dead carbon, one might hypothesize that aquatic pollen could contribute to the observed post‑1970s–1990s age-increase shifts in our seven lakes (after the ‘knot’ year identified in Table S4). However, quantitative evaluation shows that this mechanism cannot explain the magnitude of the observed radiocarbon offsets. The largest lake reservoir effect (“old‑carbon” or “dead‑carbon” effect) reported on the Tibetan Plateau is approximately 20000 ¹⁴C years [77–79]. Even under the extreme assumption that all aquatic pollen grains in our samples inherit this maximum reservoir age, the resulting offset would be only ~200 years, well within the analytical uncertainty of AMS‑¹⁴C dating (Dataset S1). Using data from Ref. [76] and assuming that all non‑terrestrial pollen (~5%) were aquatic in origin, the maximum possible offset increases to ~1000 years—still far smaller than the observed age-increase shifts in our seven lakes: 1309 years (Tanggula Lake), 1620 years (Dong Co), 2844 years (Everest Lake), 3338 years (Cangmu Co), 4939 years (Aksai Chin), 8617 years (Qiangyong Co), and 10435 years (Guozha Co) (Dataset S1). These comparisons demonstrate that the influence of aquatic pollen on radiocarbon age shifts is negligible.

All pollen-concentrated samples, including those with volumes of 400 µl and undivided ones, were sent to the Xi’an Accelerator Mass Spectrometry Center for radiocarbon (AMS-^14^C) age analysis. Of the 116 samples, 27 were not measured due to insufficient carbon content (23 samples) or faulty pretreatment experimentation (4 samples) (Dataset S1). All measured ^14^C data were calibrated to calendar ages using CALIB 8.20 with the Intcal20 dataset [80] and reported with a 2σ error.

The grain size distributions (0.01–3000 µm) of all sediment samples from Qiangyong Co and the two Aksai Chin cores were measured using a Mastersizer 3000 laser particle analyser (Malvern Panalytical, UK). Before the laser diffraction measurements, 0.1–0.2 g of each sample was pretreated with 30% H_2_O_2_ and 10% HCl to remove organic matter and carbonates. The treated samples were further dispersed with 10 ml of 10% (NaPO_3_)_6_ for 10 min in an ultrasonic bath.

**Text S3: Sediment chronology (^210^Pb dating and age models)**

The age models of sediment cores from the seven lakes were established using short-lived radionuclides ^210^Pb_ex_ and ^137^Cs (Fig. S1a–g), with the aid of an R-based *serac* code [81]. For each lake sediment core, we observed a general decrease in ^210^Pb_ex_ activity on a logarithmic scale, with two or three distinct linear trends, indicating changes in the sedimentation rate. The isotope ^137^Cs was detected throughout all lake sediment cores, with its occurrence and main peak marking the beginning and peak of nuclear weapons tests in 1955 and 1963 [81–83], respectively. Another minor peak of ^137^Cs above the 1963 peak position in the sediment cores of Everest Lake and Dong Co may represent the Chernobyl event of 1986 [83]. Based on the variation features of ^210^Pb_ex_ activity, three age model analyses—piecewise constant flux constant sedimentation rate (piecewise CFCS), constant initial concentration (CIC), and constant rate of supply (CRS) [81]—were performed on the sediment cores from Guozha Co, Aksai Chin (AKSQ-1), and Cangmu Co. Because of the non-extinction of ^210^Pb_ex_ in the bottom samples, only piecewise CFCS and CIC analyses were conducted for the Everest Lake, Tanggula Lake, and Dong Co sediment cores. Owing to the abnormal distribution of ^210^Pb_ex_ activity in the topmost 6 cm of sediment samples (discussed in the next paragraph), only piecewise CFCS and CRS analyses were conducted for Qiangyong Co. Results from different ^210^Pb-age models showed that the ages derived from the piecewise CFCS model agreed better with the 1955, 1963, and 1986 (if present) ^137^Cs time-markers. Therefore, the piecewise CFCS model result was selected as the final chronology for each lake sediment core. Overall, we observed that the sedimentation rates of lakes in the Indian monsoon climate domain were relatively higher, ranging from 1.63 to 6.85 mm yr^-1^. In contrast, sedimentation rates varied from 0.46 to 2.25 mm yr^-1^ in lakes in the westerly climate domain.

The ^210^Pb_ex_ activity in the uppermost 6 cm of sediment from Qiangyong Co was markedly lower than that of deeper layers, exhibiting a nearly constant or faintly increasing trend (Fig. S1b). Such anomalous ^210^Pb_ex_ profiles are typically attributed to sedimentary disturbances, such as sediment slumping, or to the isotope dilution effects arising from clastic particles with low excess concentrations of Pb nuclides [84,85]. Figure S2 illustrates the relationship between ^210^Pb_ex_ activity and sand content under both rapid and slow deposition regimes, as modelled by the piecewise CFCS approach (Fig. S1b). The data revealed a clear exponential decline in ^210^Pb_ex_ activity with increasing sand content and sedimentation rate, highlighting a pronounced isotope dilution effect driven by sandy clastic input in high-deposition environments. These findings suggest that the irregular ^210^Pb_ex_ distribution in the upper Qiangyong Co sediments is primarily due to the enhanced presence of coarse particles under normally accelerated depositional conditions (Fig. S2). Furthermore, the anomalous ^210^Pb_ex_ values fell within the extrapolated range of fast sedimentation rates derived from deeper sediment layers (cyan line in Fig. S1b), further supporting this interpretation. Regardless, to estimate the age of these anomalous layers, we applied the average sedimentation rate of the adjacent lower section (2.67 mm yr^-1^ in Fig. S1b). While we acknowledge that this approach may introduce some uncertainty— potential offsetting the inferred depositional ages— the impact on our central conclusion regarding the glacier melt regime shift around 1990 remains negligible. This is because sediments with anomalous ^210^Pb_ex_ values were normally deposited after 1994 (Dataset S1).

Because two lake sediment cores, AKSQ-1 and AKSQ-2, were recovered from Aksai Chin and only core AKSQ-1 was investigated for ^210^Pb and ^137^Cs activity, the chronology of AKSQ-2 was determined in two steps. First, we compared the features of the sand content in AKSQ-1 and AKSQ-2 sediments based on grain size data and paired 6 samples, which characterised the corresponding peaks and valleys of sand content between both cores to define rough dates for core AKSQ-2 (Fig. S1h). Then, with the aid of the *Bacon* package in R software, a Bayesian age model [86] was applied to fully constrain the chronology of the AKSQ-2 sediments (Fig. S1i). Note that there were discrepancies in the depths of the 6 paired samples between both sediment cores, with AKSQ-2 generally higher than AKSQ-1 (Fig. S1h). This was caused by the compaction of AKSQ-2 during its transfer to the TPESER in Beijing, whereas AKSQ-1 was sliced immediately during the fieldwork. Hence, these discrepancies do not indicate any difference in the deposition rates between AKSQ-1 and AKSQ-2, as both were located close to each other (Table S1).

**Text S4: Integrated assessment of glacier, permafrost, and vegetation contributions to pollen radiocarbon age variations**

To quantify how the processes of glaciers, permafrost, and vegetation jointly shape pollen radiocarbon age variations, we conducted an integrated two-part assessment combining (i) spatial regression modelling and (ii) temporal environmental reconstructions.

(i) We first evaluated spatial controls using multiple linear regression implemented with the R packages *relaimpo* [87] and *car* [88]. The predictor variables included the glacier and permafrost meltwater supply coefficients, and NDVI_max_, each standardised before estimating the regression coefficient but not for identifying relative importance. Variance inflation factors (VIF) were used to diagnose multicollinearity, with a VIF > 10 indicating severe predictor dependence. When collinearity was detected, we applied ridge regression using the R package *glmnet* [89] to stabilise coefficient estimates and constrain the ordinary least squares (OSL) solutions.

Because the westerly and Indian monsoon climate domains differ markedly in meteorological, cryospheric, and ecological conditions (Table S1), all analyses were conducted separately for each domain. For both domains, we evaluated two temporal intervals: post-2000 and full-period. The post-2000 interval was emphasised because the glacier area (2006–2010 [56]), permafrost area (2000–2016 [61]), and NDVI_max_ (2002–2017 [62]) datasets overlap mostly during this period, minimising temporal mismatch and enabling a robust space-for-time substitution [24]. Full-period models provide additional context and comparisons.

The multiple linear regression models showed high explanatory power (*R*^2^ = 0.86–0.99). However, VIF values > 10 revealed severe multicollinearity, particularly between the glacier meltwater supply coefficient and NDVI_max_ (Fig. S6 and Table S3), resulting in unstable OSL coefficients, disappearance of NDVI_max_ effects in monsoon models, and sign reversals in permafrost terms. Therefore, ridge regression was required to obtain physically interpretable and statistically stable coefficients.

In the westerly domain, across all models, the glacier meltwater supply coefficient emerged as the dominant positive driver of pollen radiocarbon age variations. In the post-2000 OSL models, the glacier meltwater supply coefficient (β = 0.5481, *P* = 0.0565) and NDVI_max_ (β = 0.4521, *P* = 0.0893) contributed nearly equal but opposing influences, each accounting for ~49% of the relative importance. Ridge regression confirmed these stable antagonistic effects, yielding coefficients of +0.49 for the glacier meltwater supply coefficient and −0.46 for NDVI_max_. The permafrost meltwater supply coefficient exerted only a weak negative influence (ridge coefficient = −0.04). These results indicate that glacier processes and vegetation productivity are the principal controls in westerly catchments, with minimal contributions from permafrost.

The models for the Indian monsoon domain exhibited lower explanatory power (*R*^2^ = 0.80–0.86) and stronger collinearity. The glacier meltwater supply coefficient remained a consistent positive driver (β = 4.49 and *P* = 0.0923 in post-2000 OSL). NDVI_max_ effects were masked in OLS but recovered by ridge regression as stable negative coefficients (−0.39). Permafrost contributions were context-dependent: OLS suggested negative effects (β = −3.64 and *P* = 0.1485 in post-2000 models), whereas ridge regression constrained these to weak positive coefficients (+0.21), implying that the influence of permafrost varies possibly with the balance between surface thaw, deeper thaw, and slope-collapse processes.

For both climate domains, the full-period models yielded similar patterns. Together, the spatial analyses demonstrate that glacier processes and vegetation productivity exert strong, opposing, and dominant controls on pollen radiocarbon age variations. Permafrost processes contribute variably and with substantially weaker magnitudes.

(ii) To assess temporal controls, we calculated basin-scale vegetation productivity using BCC-ESM1 historical net primary productivity (NPP) simulations from 1850 to 2014 [90]. Nearly all catchments exhibited sustained increases in NPP (Fig. S7), with statistically significant positive trends at Everest Lake (0.021 kg C m^-2^ a^-1^ per decade), Qiangyong Co (0.012), Aksai Chin (0.002), Guozha Co (0.001), Cangmu Co (0.006), and Dong Co (0.002). Only Tanggula Lake showed no significant trend. This long-term intensification implies an increase in the production of young pollen, which—if vegetation was the dominant driver—should have progressively lowered pollen radiocarbon ages.

Simultaneously, the Tibetan Plateau has experienced a persistent bipolar moisture pattern since the mid-19^th^ century, with increasing aridity in the Indian monsoon domain and increasing wetness in the westerly domain [31,32]. These divergent hydroclimatic trajectories should have produced contrasting permafrost erosion intensity and vegetation responses. However, pollen radiocarbon ages across all seven lakes exhibited coherent temporal patterns, including a shared shift from decreasing to increasing ages around the early 1990s. If vegetation or permafrost processes were the primary drivers, these temporal trajectories would differ sharply between domains. However, their coherence indicates a common forcing mechanism.

Therefore, combining the spatial and temporal evidence, we conclude that the age structure of pollen entrained in glacier ice and snow is the principal determinant of lake-deposited pollen radiocarbon ages. Vegetation and permafrost processes supply younger material that dilutes, but does not override, the glacier-derived age signal. Additional mechanistic explanations for the dilution effects of vegetation and permafrost processes are provided in the main text.

**Text S5: Trend analysis of the radiocarbon ages of pollen grains**

We first applied the lm() function in R (v4.3.3) [91] with the options “smooth = T” and “quadratic = T” to investigate the variation tendencies of the radiocarbon ages of pollen grains for all sediment samples. Before that, based on their consistency, the data from the two Aksai Chin cores were pooled into a high-time-resolution (1–15 years) sequence (Fig. S8). Tukey’s tests indicated that the radiocarbon ages of pollen grains from Everest Lake, Qiangyong Co, Tanggula Lake, Aksai Chin, and Guozha Co sediments exhibited a significantly (*P* < 0.05) nonlinear trend. The radiocarbon ages of pollen grains from Cangmu Co and Dong Co sediments were further checked for normality, non-constant variance, and autocorrelation in the linear model residues using the shapiro.test(), ncvTest(), and durbinWatsonTest() functions, respectively. The autocorrelation check suggested non-independence of residues, and hence a nonlinear trend, for the AMS-^14^C ages of pollen grains from Dong Co sediments. Although all the aforementioned tests revealed a linear tendency for the radiocarbon ages of pollen grains from Cangmu Co sediments, the correlation coefficient and root-mean-square error (RMSE) were relatively low (*R*^2^ = 0.19) and high (1082), respectively.

We then used Davies’ test with the aid of the *segmented* package [92] in R to identify where there was piecewise linear trend and, if so, potential breakpoints for each sequence of the radiocarbon ages of pollen grains (Table S4). We observed significant (*P* < 0.01) piecewise linear trends for Qiangyong Co, Aksai Chin, and Guozha Co data, with potential breakpoints being 1990, 1986, and 1992, respectively. Dong Co samples also showed a piecewise linear trend with a breakpoint of 1977, but only at a significance level of 0.1. For Everest Lake, Tanggula Lake, and Cangmu Co samples, we observed breakpoints in 1979, 1982, and 1991, respectively, although they were not significant (*P* > 0.1).

In light of the foregoing results and adhering to the principle of avoiding overfitting, we utilised the bs() function from the *splines* package [93] in R to perform quadratic (2 degrees) B-spline analyses on the radiocarbon ages of the pollen grains from each sediment core. The internal knot was determined by manually adjusting 20 years at 5-year intervals around the breakpoint suggested by Davies’ test and/or other visible turning points in the pollen radiocarbon ages for each lake sediment core. The final quadratic B-spline model was selected based on the minimum number of internal knots that achieved the highest correlation coefficient (*R*^2^ > 0.5) and the lowest *P* value (*P* < 0.05) (Table S3). Additionally, because quadratic B-spline analysis can be conducted as linear regression using the lm() function, we performed further associated nonlinearity tests (Tukey test) and regression diagnostics, including normality, non-constant variance, and autocorrelation. Consequently, the quadratic B-spline models with knots at 1990 for Qiangyong Co, 1980 for Tanggula Lake, 1920 and 1970 for Aksai Chin, 1980 for Guozha Co, 1950 for Cangmu Co, and 1975 for Dong Co, which resulted in the best fitting-correlations and passed the diagnostics, were selected. One exception to note is that the *P*-value for Tanggula samples was 0.06, which we recognised as ‘significant’ in this study because the quadratic B-spline analysis of fraction modern (Fm) for Tanggula pollen samples passed the significance level of 0.05 (Table S4). Moreover, compared to the linear regression analysis, the correlation coefficient (*R*^2^ = 0.52) and RMSE (838) in the final quadratic B-spline model of the Cangmu Co samples were significantly improved. The quadratic B-spline models ultimately show a general shift from a decreasing to an increasing trend around the 1990s for the pollen radiocarbon ages in the six lakes (Fig. 2c), consistent with the results of Davies’ test.

We also performed a quadratic B-spline analysis for the five radiocarbon ages of pollen grains from Everest Lake sediments, setting the knot at 1980 based on Davies’ test results. However, owing to the very limited number of data points, this analysis was not statistically significant (Table S3), and the results are presented for reference only.

**Supplementary Data Figures**

**Fig. S1.** Chronology of the sediments in the seven studied proglacial lakes. Figures (a–g) depict ^210^Pb and ^137^Cs activities as functions of sediment depth and age in the seven lakes. Figures (h) and (i) compare the sand contents between the two sediment cores in Aksai Chin (AKSQ-1 and AKSQ-2) and the chronology of AKSQ-2 based on 6 paired samples, corresponding to the peaks and valleys of the sand contents in AKSQ-1. (a) Everest Lake. (b) Qiangyong Co. The light grey shaded area in the left two panels highlights unusual data of ^210^Pb_ex_ and ^137^Cs not used for chronology. (c) Tanggula Lake. (d) Core1 of Aksai Chin (AKSQ-1). (e) Guozha Co. (f) Cangmu Co. (g) Dong Co. (h) Sand content in AKSQ-1 and AKSQ-2 sediment cores. (i) Core2 of Aksai Chin (AKSQ-2). All chronologies were determined using the *serac* [81] or *Bacon* [86] packages in R software (Software S1). Horizontal error bars for ^210^Pb_ex_ and ^137^Cs denote one standard error. Vertical error bars in (a–g) represent the depth range for the ^210^Pb_ex_ and ^137^Cs measurements. The red line in (i) represents the 'best' model based on the weighted mean age for each depth, bounded by 95% confidence intervals (darker grey shaded region). The three panels in (i) depict Markov Chain Monte Carlo iterations (upper left), memory (lower right), and prior (green curves) and posterior (grey histograms) distributions for the accumulation rate (upper right). All data are available in the Dataset S1.

**Fig. S2.** Relationship between ^210^Pb_ex_ activity and sand content in Qiangyong Co sediments under normal rapid and slow deposition regimes. The rapid and slow deposition regimes are defined by the piecewise CFCS model (Fig. S1b). All data are available in the Dataset S1.

**Fig. S3.** Diagram of pollen concentration in selected sediments from Guozha Co (GZC), Everest Lake (EL), Aksai Chin (AKSQ), Cangmu Co (CMC), and Dong Co (DC) proglacial lakes. All data are available in the Dataset S1.

**Fig. S4.** Common pollen types identified in selected sediments from Guozha Co, Everest Lake, Aksai Chin, Cangmu Co, and Dong Co proglacial lakes.

**Fig. S5.** Variations in the ablation area mass balance gradients (ablation gradients) with equilibrium line altitudes. (a) Schematic representation of decreasing ablation gradients with increasing equilibrium line altitudes (ELAs) from time t_0_ (black line) to t_i_ (pink line). This is revealed by our pollen radiocarbon age data and previously reported spatial [40] and temporal [41–43] distributions of glacier ablation gradients in High Mountain Asia, as depicted in (b–f). (b) Negative correlations of ablation gradients with ELAs for 2012–2016 for glaciers [40] located in the Indian monsoon (blue dots, n = 16) and westerly (red dots, n = 15; Urumqi Glacier No.1 is not included) climate domains. The current boundary between the two climate domains is represented by a dashed purple line [22]. (c–f) Examples of interdecadal decreasing ablation gradients with increasing ELAs for four representative glaciers: Urumqi Glacier No. 1 from 1982–2000 [41] to 2006–2022 [42], Ts. Tuyuksuyskiy Glacier from 1991–2007 to 2008–2023 [42], Mera Glacier from 2008–2015 to 2016–2023 [43], and Yala Glacier from 2012–2017 to 2018–2023 [42]. According to the World Glacier Monitoring Service, the Urumqi No. 1 and Ts. Tuyuksuyskiy are reference glaciers located in the westerly climate domain, and Mera and Yala are benchmark glaciers in the Indian monsoon climate domain (glacier locations are presented in (b) with larger dots). The normalised elevation was calculated as (*h*–*h*_min_)/(*h*_max_–*h*_min_), where *h* is the mid-value of each elevation band for each glacier, and *h*_min_ and *h*_max_ are the minimum and maximum mid-values of the elevation bands, respectively, for different periods. The normalised elevation was chosen to facilitate a better comparison between both periods for each glacier, considering the retreat and subsequent altitude increase of the glacier terminus. All shaded areas in the right upper panel of (b) and in (c–f) indicate 95% confidence bands.

**Fig. S6.** Environmental controls on pollen radiocarbon (AMS-^14^C) ages across seven lakes. Multiple linear (a–c, f–h, k–m, and p–r) and ridge (d–e, i–j, n–o, and s–t) regression analyses quantified the net effects of permafrost meltwater supply coefficient (permafrost), glacier meltwater supply coefficient (glacier), and vegetation production (NDVI_max_) on pollen radiocarbon ages. Partial regression plots for post-2000 (a–c) and full-period (f–h) in the westerly domain isolate each predictor’s contribution after accounting for covariation among variables, with corresponding ridge coefficient paths (d–e), and cross-validation error curves (i–j). Equivalent analyses for the Indian monsoon domain are shown in panels (k–m) and (p–r), with ridge regression diagnostics shown in (n–o) and (s–t). Together with Table S3, these results demonstrate that glacier processes and vegetation production exert dominant positive and negative controls on pollen radiocarbon age variations, whereas permafrost processes contribute weakly.

**Fig. S7.** Long‑term trends in simulated net primary productivity (NPP) across seven lake catchments from BCC‑ESM1 historical runs (1850–2014). Time‑series outputs from the BCC‑ESM1 historical experiment show broadly increasing NPP across most catchments over the past 150 years, with statistically significant positive trends at Everest Lake, Qiangyong Co, Aksai Chin, Guozha Co, Cangmu Co, and Dong Co. Each panel displays the annual NPP trajectories alongside decadal trend estimates and associated *P*-values. Data used are from reference [90].

**Fig. S8.** Pollen radiocarbon (AMS-^14^C) ages in two lake sediment cores (AKSQ-1 and AKSQ-2) of Aksai Chin. The blue dashed box highlights the consistency of the pollen radiocarbon ages between the two cores. Therefore, the data from the two cores were pooled as a high-resolution sequence. Horizontal error bars (smaller than the size of circles owing to the large scale of the pollen radiocarbon ages) indicate the two standard errors of pollen radiocarbon ages, and vertical ones indicate the depths and age ranges of lake sediments.

**Supplementary Data Tables**

**Table S1.** Sediment core locations and detailed climatic, geographic, and geologic settings of each lake catchment.

| Lake name | Coring site | | Core length (cm) | Water depth (m) | Core number | Sampling date | Catchment area (km^2^) | Lake area (km^2^) | Glacier area (km^2^) | Permafrost area (km^2^) | Median of annual maximum Normalised Difference Vegetation Index (NDVI_max_) | Glacier coverage (%) | Permafrost extent (%) | Lake supply coefficient | Glacier meltwater supply coefficient | Permafrost meltwater supply coefficient | Current regional equilibrium line altitude (ELA; m) | Mean annual precipitation (mm, 1979-2018) | Mean annual temperature (℃, 1979-2018) | | Lithology of bedrock underneath upstream glaciers | |
| --- | --- | --- | --- | --- | --- | --- | --- | --- | --- | --- | --- | --- | --- | --- | --- | --- | --- | --- | --- | --- | --- | --- |
|  | Latitude (N) | Longitude (E) |  |  |  |  |  |  |  |  |  |  |  |  |  |  |  |  |  |  |  |  |
| Everest Lake | 28°11'06" | 86°48'17" | 25 | 6 | ZF-2 | May 25, 2018 | 1.077 | 0.002 | 0.126 | 0.949 | 0.067 | 11.7 | 88.1 | 538.5 | 63.0 | 474.5 | ~5800 | 477 | -4.1 | leucogranite, amphibolite- to granulite-facies metasedimentary rocks | |  |
| Qiangyong Co | 28°53'24" | 90°13'31" | 50 | 19 | QYC-4 | August 23, 2018 | 12.0 | 0.8 | 6.6 | 4.6 | 0.159 | 55.0 | 38.3 | 15.0 | 8.3 | 5.8 | ~5800 | 379 | -3.1 | mesozoic metamorphic sandstones and slates | |  |
| Tanggula Lake | 32°55'08" | 91°58'47" | 28 | 2 | TGL-1 | June 16, 2017 | 91.9 | 6.4 | 12.8 | 71.2 | 0.356 | 13.9 | 77.5 | 14.4 | 2.0 | 11.1 | ~5700 | 475 | -3.0 | granite-porphyries and rhyolites | |  |
| Akasi Chin | 35°15'39" | 79°49'32" | 34 | 13 | AKSQ-1 | October 3, 2017 | 7992.1 | 265.0 | 688.6 | 6949.9 | 0.088 | 8.6 | 87.0 | 30.2 | 2.6 | 26.2 | ~5500 | 183 | -6.0 | metamorphic rocks including granitic gneiss, migmatites, monozogranites and schists | |  |
|  | 35°15'43" | 79°49'26" | 25 | 12 | AKSQ-2 |  |  |  |  |  |  |  |  |  |  |  |  |  |  |  |  |  |
| Guozha Co | 34°59'04" | 80°57'39" | 36 | 83 | GZC-1 | October 6, 2017 | 2726.5 | 248.4 | 543.6 | 1930.6 | 0.087 | 19.9 | 70.8 | 11.0 | 2.2 | 7.8 |  | 166 | -7.9 | metamorphic rocks including granitic gneiss, migmatites, monozogranites and schists | |  |
| Cangmu Co | 32°06'09" | 83°33'44" | 24 | 7 | CMC-4 | October 10, 2017 | 2723.0 | 115.2 | 99.2 | 1294.7 | 0.187 | 3.6 | 47.5 | 23.6 | 0.9 | 11.2 | ~5900 | 206 | 1.5 | amphibolite-facies metamorphic rocks and granite | |  |
| Dong Co | 32°09'51" | 84°44'03" | 29 | 4 | DC-3 |  | 5999.0 | 117.0 | 30.7 | 2056.8 | 0.181 | 0.5 | 34.3 | 51.3 | 0.3 | 17.6 |  | 300 | -0.1 | granitoids, schists, quartize, and argillite | |  |

The table shows the coring site (latitude and longitude), core length, water depth of the coring site, sampling date, catchment area, lake area, glacier area, permafrost area, median of annual maximum Normalised Difference Vegetation Index (NDVI_max_), glacier coverage, permafrost extent, lake supply coefficient, glacier and permafrost meltwater supply coefficients, mean annual precipitation, mean annual temperature, and lithology of bedrock beneath upstream glaciers. Glacier and permafrost coverage were expressed as the ratio of the glacier and permafrost areas to the catchment area, respectively. The lake supply and glacier and permafrost meltwater supply coefficients were calculated as the area ratios of the catchments, upstream glaciers, and upstream permafrost to the lakes, respectively. Data on glacier area, permafrost area, NDVI_max_, equilibrium line altitude, and lithology of bedrock were obtained from references [56–59], [61], [62], [40,59,60], and [65–70], respectively. The mean annual precipitation and temperature for 1979–2018 were calculated using reported data from reference [55].

**Table S2.** Average radiocarbon (AMS-^14^C) ages of pollen grains for each lake, alongside the mean radiocarbon ages of soil organic matter in the surface (0–10 cm) and deep (30–50 cm) layers. Differences among these age groups were evaluated using one-way ANOVA, followed by post-hoc LSD tests.

| Lake/Name | Pollen AMS ^14^C age (cal yr BP) | | | Climate regime | One-way ANOVA with post-hoc LSD tests (*P* value) | | | | | | |
| --- | --- | --- | --- | --- | --- | --- | --- | --- | --- | --- | --- |
|  | Number | Average | 1σ |  | Everest Lake | Qiangyong Co | Tanggula Lake | Aksai Chin | Guozha Co | Cangmu Co | Dong Co |
| Everest Lake | 5 | 14526 | 864 | Indian monsoon |  |  |  |  |  |  |  |
| Qiangyong Co | 11 | 6786 | 795 |  | 0.000 |  |  |  |  |  |  |
| Tanggula Lake | 9 | 3535 | 401 |  | 0.000 | 0.000 |  |  |  |  |  |
| Aksai Chin | 18 | 19818 | 691 | westerly | 0.000 | 0.000 | 0.000 |  |  |  |  |
| Guozha Co | 9 | 16943 | 1229 |  | 0.024 | 0.000 | 0.000 | 0.000 |  |  |  |
| Cangmu Co | 15 | 7702 | 321 |  | 0.000 | 0.227 | 0.000 | 0.000 | 0.000 |  |  |
| Dong Co | 22 | 6637 | 219 |  | 0.000 | 0.833 | 0.000 | 0.000 | 0.000 | 0.097 |  |
| Surface soil (0–10 cm) | 21 | 203 | 72 | both | 0.000 | 0.000 | 0.000 | 0.000 | 0.000 | 0.000 | 0.000 |
| Deep soil (30–50 cm) | 30 | 2567 | 298 |  | 0.000 | 0.000 | 0.183 | 0.000 | 0.000 | 0.000 | 0.000 |

Statistical significance was set at *P* < 0.05. Radiocarbon age data for soil organic matter in the surface (0–10 cm) and deep (30–50 cm) layers were compiled from references [29] and [28], respectively.

**Table S3.** Regression results for environmental controls on pollen radiocarbon ages. Summary of multiple linear and ridge regression analyses assessing the influence of permafrost and glacier meltwater supply coefficients, and vegetation production (NDVI_max_) on pollen radiocarbon ages in lakes located in the westerly (4 lakes) and Indian monsoon (3 lakes) domains, using both post-2000 and full-period datasets

| Period | Region | Analyses | | | | | | | |
| --- | --- | --- | --- | --- | --- | --- | --- | --- | --- |
| Post-2000 | westerly (4 lakes) |  | Multiple linear regression (*R*^2^ = 0.9924, Adjusted *R* ^2^= 0.9848; *P* = 0.0011) | | | | | | Ridge regression |
|  |  |  | Estimate (Standardized regression coefficient) | Standard error | t value | Pr (> \|t\|) | Relative importance (%) | Variance inflation factors (VIF) | Ridge-penalized coefficients |
|  |  | Intercept | 0.0000 | 0.0467 | 0.000 | 1.0000 |  |  | 0.0000 |
|  |  | Permafrost meltwater supply coefficient | -0.0394 | 0.0516 | -0.763 | 0.5008 | 1.1180 | 1.0501 | -0.0404 |
|  |  | Glacier meltwater supply coefficient | **0.5481** | 0.1811 | 3.027 | **0.0565·** | **49.3485** | 12.9143 | **0.4908** |
|  |  | NDVI_max_ | **-0.4521** | 0.1823 | -2.48 | **0.0893·** | **48.7718** | 13.0882 | **-0.4614** |
|  | Indian monsoon (3 lakes) |  | Multiple linear regression (*R*^2^ = 0.8604, Adjusted *R*^2^ = 0.7907; *P* = 0.0195) | | | | | | Ridge regression |
|  |  |  | Estimate (Standardized regression coefficient) | Standard error | t value | Pr (> \|t\|) | Relative importance (%) | Variance inflation factors (VIF) | Ridge-penalized coefficients |
|  |  | Intercept | 0.0000 | 0.1729 | 0.000 | 1.0000 | NA | NA | 0.0000 |
|  |  | Permafrost meltwater supply coefficient | -3.6400 | 2.0380 | -1.787 | 0.1485 | NA | NA | 0.2071 |
|  |  | Glacier meltwater supply coefficient | 4.4900 | 2.0380 | 2.204 | 0.0923· | NA | NA | **0.2395** |
|  |  | NDVI_max_ | NA | NA | NA | NA | NA | NA | **-0.3889** |
| Full period | westerly (4 lakes) |  | Multiple linear regression (*R*^2^ = 0.8820, Adjusted *R*^2^ = 0.8761; *P* < 0.0001) | | | | | | Ridge regression |
|  |  |  | Estimate (Standardized regression coefficient) | Standard error | t value | Pr (> \|t\|) | Relative importance (%) | Variance inflation factors (VIF) | Ridge-penalized coefficients |
|  |  | Intercept | 0.0000 | 0.0440 | 0.000 | 1.0000 |  |  | 0.0000 |
|  |  | Permafrost meltwater supply coefficient | 0.0936 | 0.0483 | 1.939 | **0.0572·** | 7.2171 | 1.1834 | 0.1014 |
|  |  | Glacier meltwater supply coefficient | **0.5102** | 0.1458 | 3.498 | **0.0009***** | **40.9772** | 10.8116 | **0.4519** |
|  |  | NDVI_max_ | **-0.3988** | 0.1457 | -2.737 | **0.0081**** | **40.0013** | 10.7900 | **-0.4131** |
|  | Indian monsoon (3 lakes) |  | Multiple linear regression (*R*^2^ = 0.8027, Adjusted *R*^2^ = 0.7848; *P* < 0.0001) | | | | | | Ridge regression |
|  |  |  | Estimate (Standardized regression coefficient) | Standard error | t value | Pr (> \|t\|) | Relative importance (%) | Variance inflation factors (VIF) | Ridge-penalized coefficients |
|  |  | Intercept | 0.0000 | 0.0928 | 0.000 | 1.0000 | NA | NA | 0.0000 |
|  |  | Permafrost meltwater supply coefficient | -1.6790 | 0.7086 | -2.37 | 0.0270 * | NA | NA | 0.2803 |
|  |  | Glacier meltwater supply coefficient | 2.5310 | 0.7086 | 3.572 | 0.0017** | NA | NA | **0.3062** |
|  |  | NDVI_max_ | NA | NA | NA | NA | NA | NA | **-0.3550** |

Significance codes: *** *P* < 0.001, ** *P* < 0.01, * *P* < 0.05; **·** *P* < 0.1.

The NA value indicates that NDVI_max_ is perfectly collinear with the glacier meltwater supply coefficient (Fig. S6).

**Table S4.** Correlation coefficients (*R*^2^) and *P* values for the trend analysis of the pollen radiocarbon (AMS-^14^C) ages in each lake.

| Lake | Davies’ test for AMS-^14^C ages of pollen grains | |  | Quadratic B-spline for AMS-^14^C ages of pollen grains | | | |  | Quadratic B-spline for fraction modern (F_m_) | | | |  |
| --- | --- | --- | --- | --- | --- | --- | --- | --- | --- | --- | --- | --- | --- |
|  | Break points | *P* |  | Knots | *R*^2^ | *P* | RMSE |  | Knots | *R*^2^ | *P* | RMSE |  |
| Everest Lake | 1979 | 0.211 |  | 1980 | 0.88 | 0.425 | 588.96 |  | 1980 | 0.75 | 0.607 | 0.0176 |  |
| Qiangyong Co | 1990 | 0.005 |  | 1990 | 0.84 | 0.004 | 1002.68 |  | 1990 | 0.83 | 0.004 | 0.0500 |  |
| Tanggula Lake | 1982 | 0.155 |  | 1980 | 0.74 | 0.063 | 577.81 |  | 1980 | 0.78 | 0.042 | 0.0346 |  |
| Aksai Chin | 1986 | 0.004 |  | 1920, 1970 | 0.87 | 0.000 | 1043.80 |  | 1920, 1970 | 0.89 | 0.000 | 0.0124 |  |
| Guozha Co | 1992 | 0.005 |  | 1980 | 0.92 | 0.003 | 970.70 |  | 1980 | 0.91 | 0.005 | 0.0124 |  |
| Cangmu Co | 1991 | 0.193 |  | 1950 | 0.52 | 0.041 | 837.50 |  | 1950 | 0.53 | 0.036 | 0.0429 |  |
| Dong Co | 1977 | 0.078 |  | 1975 | 0.56 | 0.002 | 663.61 |  | 1975 | 0.58 | 0.001 | 0.0364 |  |

Statistical significance was set at *P* < 0.05.

**SI References**

55. He J, Yang K, Wang T *et al.* The first high-resolution meteorological forcing dataset for land process studies over China. *Sci Data* 2020; **7**: 25.
56. Guo W, Liu S, Xu J *et al.* The second Chinese glacier inventory: data, methods and results. *J Glaciol* 2015; **61**: 357–372.
57. Qiao B, Zhu L. Differences and cause analysis of changes in lakes of different supply types in the north-western Tibetan Plateau. *Hydrol Process* 2017; **31**: 2752–2763.
58. Qiao B, Zhu L, Wang J *et al.* Estimation of lakes water storage and their changes on the northwestern Tibetan Plateau based on bathymetric and Landsat data and driving force analyses. *Quat Int* 2017; **454**: 56–67.
59. Spiess M, Schneider C, Maussion F. MODIS-derived interannual variability of the equilibrium-line altitude across the Tibetan Plateau. *Ann Glaciol* 2016; **57**: 140–154.
60. Duan K, Yao T, Wang N *et al.* Changes in equilibrium-line altitude and implications for glacier evolution in the Asian high mountains in the 21st century. *Sci China Earth Sci* 2022; **65**: 1308–1316.
61. Obu J, Westermann S, Bartsch A *et al*. Northern Hemisphere permafrost map based on TTOP modelling for 2000–2016 at 1 km^2^ scale. *Earth Sci Rev* 2019; **193**: 299-316.
62. Cao B, Zhang T, Wu Q *et al*. Permafrost zonation index map and statistics over the Qinghai-Tibet Plateau based on field evidence. *Permafr Periglac Process* 2019; **30**: 178-194.
63. Ding M, Zhang Y, Liu L *et al*. The relationship between NDVI and precipitation on the Tibetan Plateau. *J Geogr Sci* 2007; **17**: 259-268.
64. Song C, Huang B, Richards K *et al.* Accelerated lake expansion on the Tibetan Plateau in the 2000s: Induced by glacial melting or other processes? *Water Resour Res* 2014; **50**: 3170–3186.
65. Luo R, Cao J, Liu G *et al.* Characteristics of the subglacially-formed debris-rich chemical deposits and related subglacial processes of Qiangyong Glacier, Tibet. *J Geogr Sci* 2003; **13**: 455–462.
66. Liu C, Wang RC, Wu FY *et al.* Spodumene pegmatites from the Pusila pluton in the higher Himalaya, South Tibet: Lithium mineralization in a highly fractionated leucogranite batholith. *Lithos* 2020; **358–359**: 105421.
67. Lu L, Qin Y, Li ZF *et al.* Diachronous closure of the Shuanghu Paleo-Tethys Ocean: Constraints from the Late Triassic Tanggula arc-related volcanism in the East Qiangtang subterrane, Central Tibet. *Lithos* 2019; **328–329**: 182–199.
68. Sundell KE, Taylor MH, Styron RH *et al.* Evidence for constriction and Pliocene acceleration of east-west extension in the North Lunggar rift region of west central Tibet. *Tectonics* 2013; **32**: 1454–1479.
69. Volkmer JE, Kapp P, Guynn JH *et al.* Cretaceous-Tertiary structural evolution of the north central Lhasa terrane, Tibet. *Tectonics* 2007; **26**: TC6007.
70. Matte P, Tapponnier P, Arnaud N *et al.* Tectonics of Western Tibet, between the Tarim and the Indus. *Earth Planet Sci Lett* 1996; **142**: 311–330.
71. Du Y, Luo C, Chen F *et al.* Water depth and transparency drive the quantity and quality of organic matter in sediments of Alpine Lakes on the Tibetan Plateau. *Limnol Oceanogr* 2022; **67**: 1959–1975.
72. Wang F, Qian N, Zhang Y *et al.* *Pollen Flora of China*. Beijing: Science Press, 1995.
73. Tang L, Mao L, Shu J *et al. Atlas of Quaternary pollen and spores in China*. Singapore: Springer Nature, 2020.
74. Grimm E. *Tilia Version 2.6.1*. Illinois State Museum: Illinois; 2019.
75. Cui A, Wang M, Lu H *et al.* Seasonal Climatic Variations Inferred From Pollen in a Laminated Glacier in the Southeastern Tibetan Plateau. *Earth Space Sci* 2022; **9**: e2022EA002581.
76. Ma Q, Zhu L, Ju J *et al.* A modern pollen dataset from lake surface sediments on the central and western Tibetan Plateau. *Earth Syst Sci Data* 2024; **16**: 311–320.
77. Hou J, D’Andrea WJ, Liu Z. The influence of ^14^C reservoir age on interpretation of paleolimnological records from the Tibetan Plateau. *Quat Sci Rev* 2012; **48**: 67–79.
78. Mischke S, Weynell M, Zhang C *et al.* Spatial variability of ^14^C reservoir effects in Tibetan Plateau lakes. *Quat Int* 2013; **313–314**: 147–155.
79. Zhang J, Ma X, Qiang M *et al.* Developing inorganic carbon-based radiocarbon chronologies for Holocene lake sediments in arid NW China. *Quat Sci Rev* 2016; **144**: 66–82.
80. Reimer PJ, Austin WEN, Bard E *et al.* The IntCal20 Northern Hemisphere Radiocarbon Age Calibration Curve (0–55 cal kBP). *Radiocarbon* 2020; **62**: 725–757.
81. Bruel R, Sabatier P. serac: an R package for ShortlivEd RAdionuclide chronology of recent sediment cores. *J Environ Radioact* 2020; **225**: 106449.
82. Appleby PG. Chronostratigraphic techniques in recent sediments. In: William ML and Smol JP (ed.). *Tracking environmental change using lake sediments: Basin Analysis, Coring, and Chronological Techniques.* Dordrecht: Kluwer Academic Publishes, 2001, 171–203.
83. Lan J, Wang T, Chawchai S *et al.* Time marker of ^137^Cs fallout maximum in lake sediments of Northwest China. *Quat Sci Rev* 2020; **241**: 106413.
84. Arias-Ortiz A, Masqué P, Garcia-Orellana J *et al.* Reviews and syntheses: ^210^Pb-derived sediment and carbon accumulation rates in vegetated coastal ecosystems – setting the record straight. *Biogeosciences* 2018; **15**: 6791–6818.
85. Wolfe AP, Miller GH, Olsen CA *et al.* Geochronology of high latitude lake sediments. In: Smol, JP (ed.). *Long-term environmental change in Arctic and Antarctic lakes*. Netherlands: Springer, 2004, 19–52.
86. Maarten B, Christen JA. Flexible paleoclimate age-depth models using an autoregressive gamma process. *Bayesian Anal* 2011; **6**: 457–474.
87. Groemping U. Relative Importance for Linear Regression in R: The Package relaimpo. *J Stat Softw* 2006; **17**: 1–27.
88. Fox J, Weisberg S, Adler D *et al*. Package ‘car’. Vienna: R foundation for statistical computing; 2012.
89. Friedman J, Hastie T, Tibshirani R et al. Package ‘glmnet’. Vienna: R foundation for statistical computing; 2021.
90. Zheng Z. Global vegetation productivity data simulated by BCC-ESM1 during 1850–2014. Beijing: National Tibetan Plateau Data Center; 2022.
91. R Core Team. R: A language and environment for statistical computing. Vienna: R foundation for statistical computing; 2024.
92. Muggeo VM. Segmented: an R package to fit regression models with broken-line relationships. *R news* 2008; **8**: 20–25.
93. Bates M, Venables B. Package 'splines'. Vienna: R foundation for statistical computing; 2024.

**Dataset S1** (separate table file). **All proglacial lake sediment and glacier ablation gradient data.**

**Software S1** (separate text file). **Code utilised for chronology and mathematical analyses.**
